# The after-hours circadian mutant has reduced phenotypic plasticity in behaviors at multiple timescales and in sleep homeostasis

**DOI:** 10.1038/s41598-017-18130-2

**Published:** 2017-12-19

**Authors:** Silvia Maggi, Edoardo Balzani, Glenda Lassi, Celina Garcia-Garcia, Andrea Plano, Stefano Espinoza, Liudmila Mus, Federico Tinarelli, Patrick M. Nolan, Raul R. Gainetdinov, Fuat Balci, Thierry Nieus, Valter Tucci

**Affiliations:** 1 Department of Neuroscience and Brain Technologies – Genetics and Epigenetics of Behaviour - Istituto Italiano di Tecnologia, via Morego, 30, 16163 Genova, Italy; 2 Faculty of Biology, Medicine and Health, University of Manchester, Manchester, M13 9PL UK; 3 MRC Harwell, Harwell Science and Innovation Campus, Oxfordshire, OX11 0RD UK; 4 Koç University, Department of Psychology, Istanbul, Turkey; 5 Institute of Translational Biomedicine, St. Petersburg State University, St. Petersburg, Russia; 6 Skolkovo Institute of Science & Technology, Skolkovo, Moscow, Russia

## Abstract

Circadian clock is known to adapt to environmental changes and can significantly influence cognitive and physiological functions. In this work, we report specific behavioral, cognitive, and sleep homeostatic defects in the after hours (*Afh*) circadian mouse mutant, which is characterized by lengthened circadian period. We found that the circadian timing irregularities in *Afh* mice resulted in higher interval timing uncertainty and suboptimal decisions due to incapability of processing probabilities. Our phenotypic observations further suggested that *Afh* mutants failed to exhibit the necessary phenotypic plasticity for adapting to temporal changes at multiple time scales (seconds-to-minutes to circadian). These behavioral effects of *Afh* mutation were complemented by the specific disruption of the Per/Cry circadian regulatory complex in brain regions that govern food anticipatory behaviors, sleep, and timing. We derive statistical predictions, which indicate that circadian clock and sleep are complementary processes in controlling behavioral/cognitive performance during 24 hrs. The results of this study have pivotal implications for understanding how the circadian clock modulates sleep and behavior.

## Introduction

Twenty four hour cycles of specific transcriptional-translational feedback loops (TTFLs) in the suprachiasmatic nucleus (SCN) of the hypothalamus drive circadian rhythms of organisms^1^. However, how these TTFLs impact behavioral/cognitive functions and the homeostatic regulation of sleep remains an open fundamental question in circadian biology^2^.

The circadian clock is a system that is characterized by an extraordinary phenotypic plasticity; for example, circadian rhythms adapt to changes in daily light due to the seasonal earth’s rotation^3,4^ and/or to the availability of food supply^5,6^. In nocturnal animals, such as mice, this phenomenon is displayed by the compression or decompression of the length of the active phase (α index) as the dark phase shortens or lengthens, respectively^3^. Several lines of evidence have shown that the circadian molecular clock regulates the onset and the offset of the active phase^3^ and that *Per2* clock gene plays a crucial role in phase delay^7^. Moreover, PER2 has a fundamental role in regulating food-entrainment^8^, sleep homeostasis^9^, and short-interval timing behaviors^10^.

Operating at a different timescale than the circadian clock, ‘interval timing’ refers to a clock system that manifests at seconds-to-minutes-long intervals and governs behavioral/cognitive processes such as short-lived behavioral responses, attention, decision-making, and memory^11^. In a sense, if the circadian clock operates as the conventional 24 hr watch, interval timing operates as a stopwatch. The interval timing system, which very much depends on the healthy functioning of striatum^12^, is also characterized by a certain degree of phenotypic plasticity. For instance, short behavioral responses can be flexibly parameterized to match the temporal statistics of the environment and in doing so rely on an endogenous timing uncertainty that, ultimately, can affect adaptive decision-making^11,13^.

The long-standing idea of a cross-talk between circadian clock and sleep has received a renewed interest with the availability of several circadian mouse models. Interestingly, both circadian clock and sleep were shown to modulate timing in humans^14^ and mice^15,16^. We recently reported that a novel, mostly cell-nonautonomous^17^, circadian axis that accelerates circadian clock in mice, is also responsible for a parallel acceleration of interval timing behaviors and disruption of the sleep homeostasis^15^. The hypothesis that circadian clock and sleep are regulators of short-interval behaviors is still in its infancy^18^ and most of the main circadian mouse models with the potential to provide insights toward the understanding of the related mechanisms remain to be studied.

In this work, we studied the after hours (*Afh*) mutant mice, which exhibit a lengthening of the circadian period. The *Afh* mutation, an A > T transversion in the *Fbxl3* gene, results in a Cys^358^Ser substitution within the F-box protein mediating CRY target ubiquitination and degradation. In effect, this delays the CRY-mediated negative feedback and increases CRY stability in *Afh* mutants^19^. Consequently, the expression of other core clock elements is also altered in homozygous mutants (i.e., *Per2*). The interaction between PER and CRY is fundamental in mediating the transcriptional repression of the Clock-Bmal1 positive loop^20^. The role of *Fbxl3* as a fundamental regulator of CRY protein has been confirmed by a complementary work on a different mouse model, *overtime* ^21^. The *Afh* mutation reduces circadian oscillations at the single cell level outside the SCN, resulting in alterations of metabolism^22^. Moreover, the *Afh* mutants present abnormal responses to light input and this particular sensitivity to light influences the mode in which their circadian clock system responds to light perturbations^22^, and can potentially impact the behavioral responses of the mouse line.

Mutants exhibited abnormal timing behaviors at multiple timescales: at hourly scale while anticipating meals, and in behavioral performance that depended on temporal information processing at seconds scale. Specifically, we found the lengthening of the circadian periodicity of work-for-food behaviors in *Afh* mutants that was accompanied by their inability to compress the length of activity in response to the shortening of the dark phase. Interval timing and decision-making were also altered during the regular light-dark schedule in *Afh* homozygous mutants. In addition, mutants exhibited abnormal sleep homeostasis. These behavioral characterizations were complemented by our findings that showed irregular *Per2* in relevant brain areas outside the SCN, including striatum.

## Results

### Circadian Timescale

#### The *Afh* mutants do not change daily activity in response to changing light-dark cycle lengths

**Figure 1:**
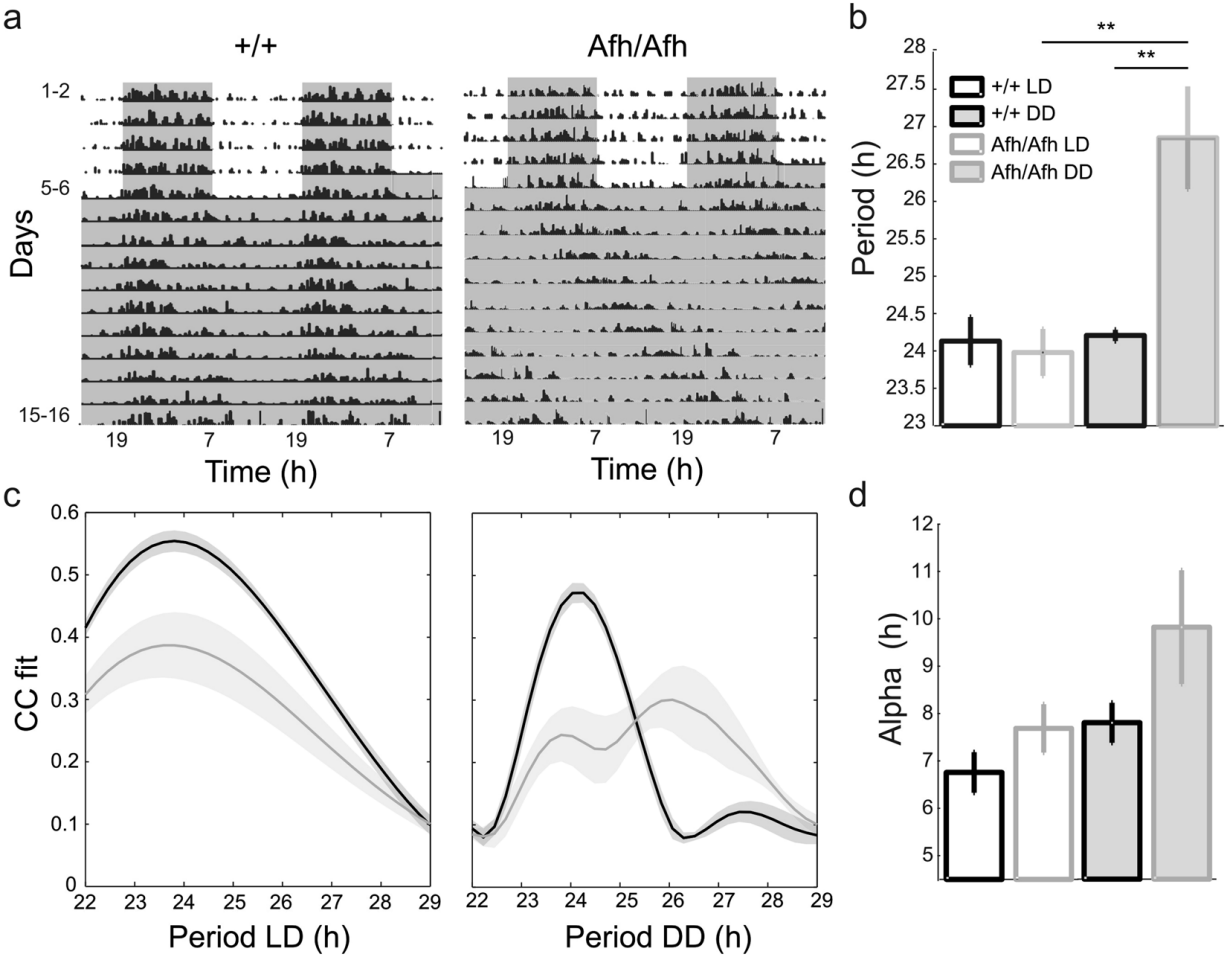
*Afh* mutants show lengthening of work-for-food circadian period. (**a**) Double plotted actograms of the nose-poke activity for a wild type (left panel) and the *Afh* homozygous mouse (right panel). The gray shadowed region represents the dark phase in LD condition and is followed by DD. Vertical black bars describe bouts of nose-poking activity for 15 min. bin. (**a**) Mean ± SEM period during LD (white histogram) and DD (gray histogram) for +/+ (n = 6; LD: 24.1 ± 0.32, DD: 24.2 ± 0.07) and *Afh*/*Afh* (n = 6; LD: 23.9 ± 0.31, DD: 26.8 ± 0.68) mice. (**p < 0.005 One-way ANOVA). (**c**) A measure of the period strength for wild type (black line) and homozygous (gray line) mutant mice in LD (left panel) and DD (right panel) phases. Mean ± SEM distributions of the correlation coefficient between periodic fit and actograms for increasing values of period. (**c**) The alpha represents the length of the segment of the daily activity.

We tested all mice while performing work-for-food behavioral tasks in their home-cages^23^, 24 hr a day for several days without any interruptions (see Methods). The circadian profile of each mouse activity was derived from the number of nose pokes emitted to retrieve food pellets. We observed that the latter behavior of *Afh* mutants mimicked the wheel-running activity profile previously reported as consequence of the *Afh* mutation^19^. *Afh* homozygous mice showed a significant lengthening of their internal clock in dark-dark (DD) condition compared to the wild-type mice (Fig. 1a,b). There was reduced strength and higher variability of correlation coefficient (CC) between *Afh* nose-poke activity and periodicity under light-dark (LD) condition (Fig. 1c). The same observations were previously reported for wheel-running behavior^19^. Moreover, in *Afh* mutants the length of the active phase α increased in DD, suggesting decompression of their activity in absence of external light-dependent oscillators (Fig. 1d).

**Figure 2:**
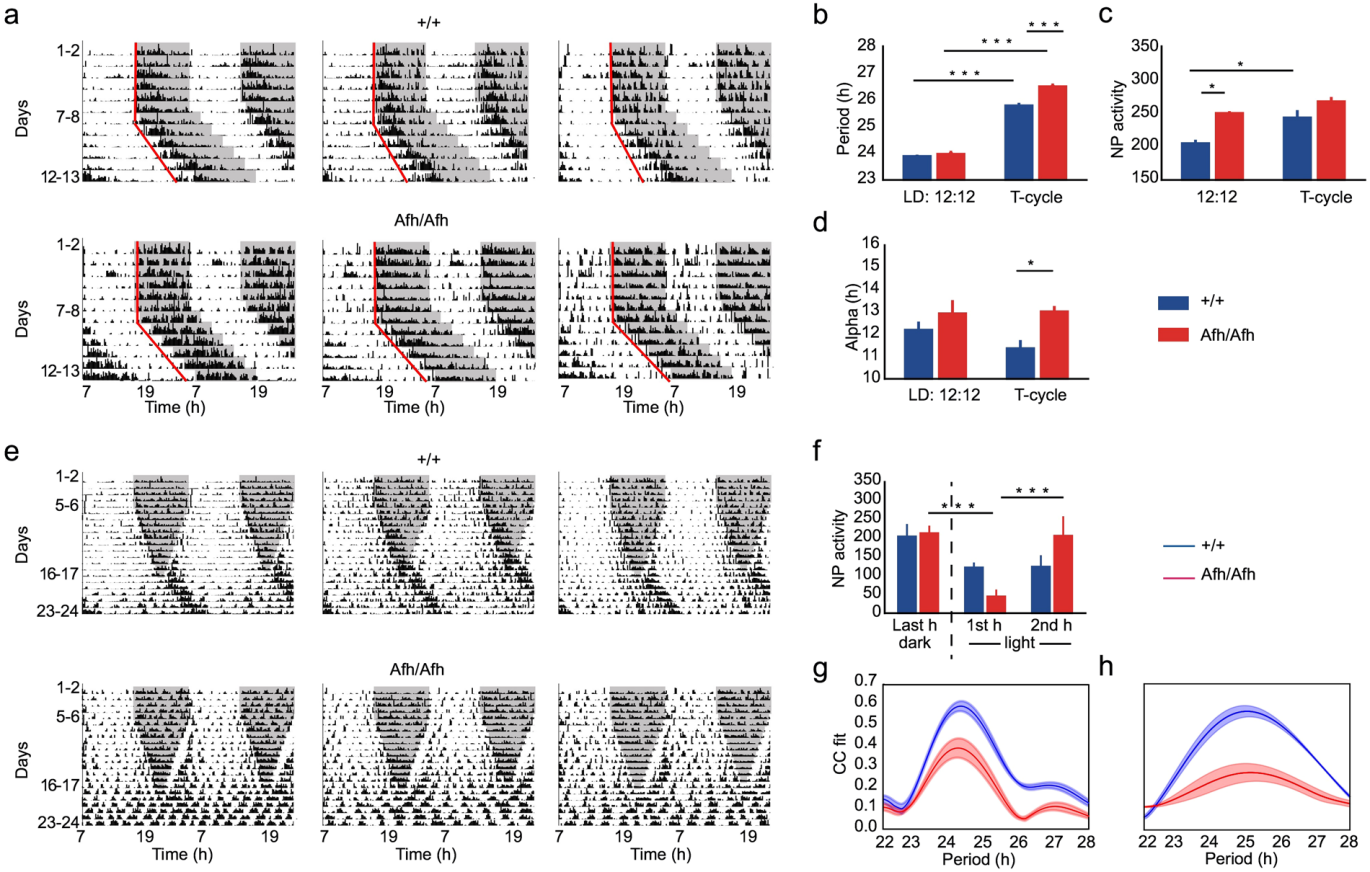
T-cycle and dark phase reduction reveals lack of phenotypic plasticity in circadian behavior in *Afh* mutants. Representative double-plotted actograms of nose poking activity for three wild-type (upper panels) and three *Afh* (lower panels) mice (**a**). Nose pokes were collected in 15 minutes bins and plotted as black bars. Dark phase is marked with a shaded region in both 12:12 light dark schedule and T-cycle. Mean + SEM of period (**b**), nose pokes (**c**) and alpha phase (**d**) are presented for LD and T-cycle conditions. Blue bars are for wild-type mice (n = 6) and red bars for *Afh* (n = 6) homozygous mutants. Examples of double-plotted actograms for wild-type (upper panels) and *Afh* (lower panels) mice (**e**). Dark phase is marked with a shaded region. Fixed LD 12:12 schedule runs is shown for 5–6 days, then all mice undergo a progressive reduction of the dark phase over 12 days. In particular, each consecutive dark phase during these 12 days is reduced by an hour (30 minutes at the beginning and 30 minutes at the end of the dark phase). (**f**) Mean + SEM of nose poke activity during last hour of the dark phase and the first two hours of the light phase are presented for wild-type mice in blue and for *Afh* in red. Pearson Correlation coefficient between a fitted sinusoidal curve and nose poke activity for a range of periodicities varying between 22 and 28 hours during dark phase reduction (**g**) and constant light (**h**) phase. Solid lines represent mean and shaded regions represent mean ± SEM. Blue lines are always associated with wild-type and red with *Afh*. *p < 0.05, ***p < 0.001, two way ANOVA with repeated measure post hoc test, Bonferroni corrected.

Thus, we tested whether matching the external daily LD oscillators with the internal clock of mutants would restore the physiological circadian rhythm in these mice. To this end, we tested all mice in a 26.5 hr T-cycle. With this long T-cycle asset, wild-type mice lengthened their circadian period but not enough to entrain to the T-cycle, as the latter cycle was longer than their internal clock (Fig. 2a, upper panels; Fig. 2b). However, as we expected, *Afh* homozygous mice lengthened their period and exhibited the entrainment to the longer T-cycle (Fig. 2a, lower panels: Fig. 2b). Although the entrainment and circadian period in *Afh* mutants fit the long T-cycle, some striking abnormalities emerged in their activity. *Afh* homozygous mice, which had an overall higher nose poking activity compared to wild-type mice (Fig. 2c in LD:12:12), showed no changes in nose pokes (Fig. 2c) or α (Fig. 2d) when shifting to T-cycle. The absence of variation between the two different light conditions suggests a deficit in the regulatory mechanisms of the two oscillators that govern the compression and decompression of the activity with changing environmental light conditions^3,24^.

#### *Afh* mutants fail to adapt their daily activity to gradually changing dark phase

The circadian pacemaker controls the onset and the offset of the animals’ activity, a phenomenon that is thought to be regulated by a dual oscillator structure that, in nocturnal animals, leads to onset of activity around the beginning of the dark phase and offset of activity around the beginning of light phase^3^.

We tested the adaptability of mice in terms of shifts in the onset/offset of their daily activity by subjecting a new cohort of mice to a progressive reduction of the dark phase over several days. In this variable light-dark condition, wild-type mice exhibited a gradual adaptation manifested by a continuing compression of their α activity. These mice adjusted the onset and the offset of their activity by aligning them with the beginning and the end of the dark phase, respectively (Fig. 2e, upper panels). Nevertheless, when the dark phase was eventually too short and they entered in the light-light (LL) condition, as expected all wild-type mice showed a lengthening of their period. Interestingly, while *Afh* mutants were able to adjust the onset of their activity with the shift of the dark period, they failed to adjust the termination of their activity. Visual inspection of Fig. 2e (lower panels) reveals a clear overflowing of the α activity as the dark phase was shortened, although this is partially covered by a brief masking effect due to onset of the light phase. The effect of light masking lasts approximately one hour and is followed by an increase in nose pokes in *Afh* mutants (Fig. 2f). Moreover, upon entering the LL condition, all mutants showed arrhythmic behavior, as demonstrated also by the low amplitude of their rhythm compared to wild-type mice (Fig. 2g,h).

### Hour timescale

#### *Afh* mutants failed to develop food anticipatory behavior

In order to address the ecological aspects of how mice adjust temporal processes at an hourly scale, we investigated the development of anticipatory behaviors of mice when food was restricted to specific periods of the day. This manipulation increases the ecological validity of the corresponding function since, for instance, in searching for food, it is adaptive and critical to predict the hours-long periods during which prey/resources become available.

**Figure 3:**
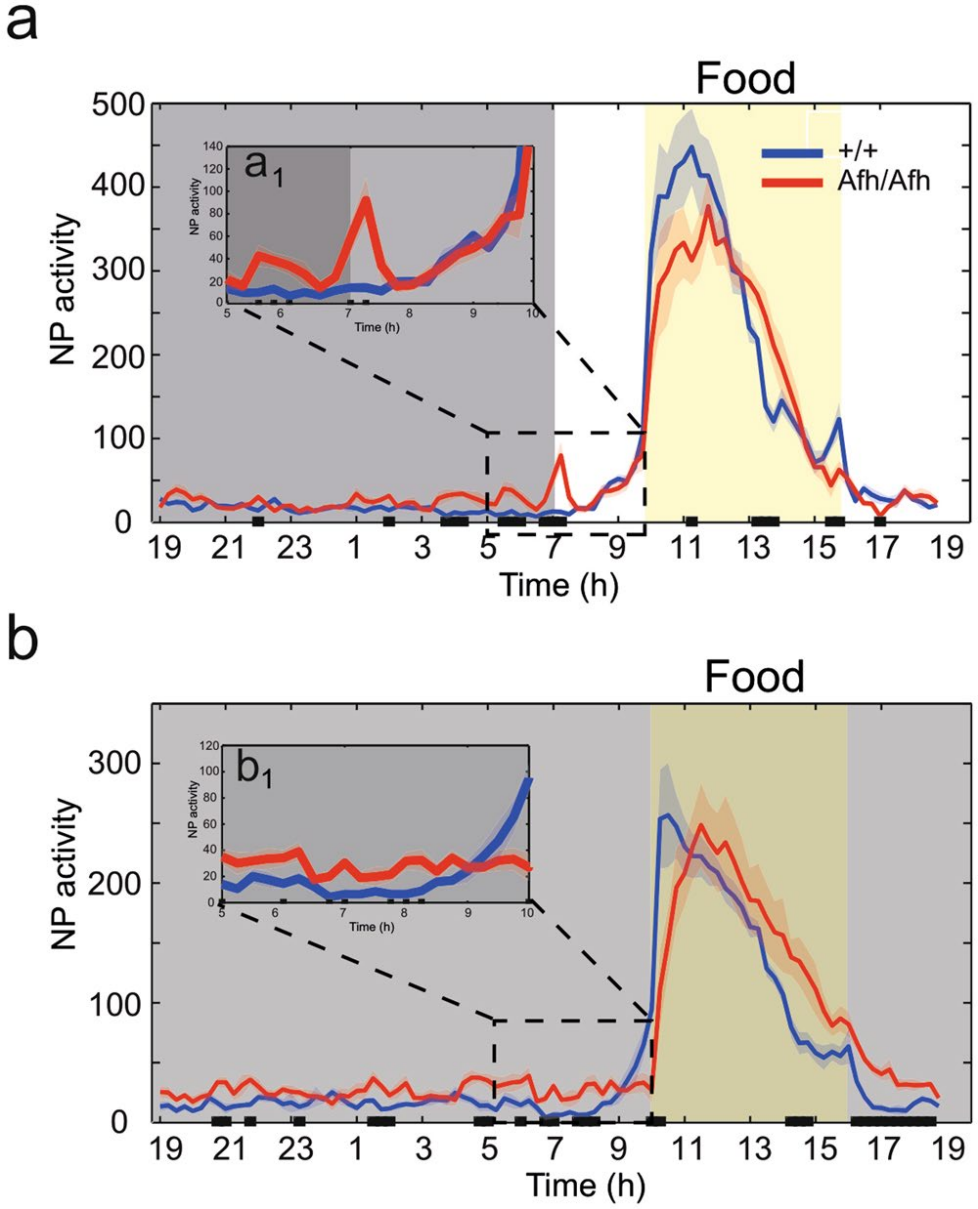
*Afh* mutants do not exhibit food anticipatory activity. (**a**) Distribution of nose pokes, Average ± SEM, over 24 h in LD (n = 6 wild-type, n = 6 *Afh*). NP activity was quantified with 15 min time bins. Grey shaded regions indicate dark periods and yellow shaded regions indicate the time when food was available. Nose poking activity is averaged across 10 days during LD (**a**) and 7 days during DD (**b**) for the mutant mice (red lines) and wild-type littermate controls (blue lines). The inset show magnification of a selected time windows (5:00 to 10:00) for the LD (a_1_) and DD (b_1_). The black squares at the bottom of each graphs identified the intervals with significant genotype difference (p < 0.05, one-way Anova).

We tested whether mutants could predict the time of feeding and show food anticipatory activity (FAA). *Afh* and wild-type mice were subjected to a fixed daily food restriction protocol in which food was available only between 10:00 and 16:00 hrs during LD and DD condition (see Methods). In LD, *Afh* homozygous mice showed a significant peak of nose poking activity between 07:00 and 8:00, after the light on, while this peak was not present in wild-type mice (Fig. 3a,a_1_). Interestingly, the 7:00–8:00 hrs peak was not present in DD and mutants failed to show food anticipatory behavior (Fig. 3b,b_1_). This suggests the occurrence of associative learning in *Afh* mutants. Indeed, *Afh* mutants successfully associated food with the onset of the light phase in the LD paradigm; however, compared to the wild-type mice, they failed to learn the time of food availability in DD, as they did not show FAA in absence of light cues (this was not the case for the wild-type mice; Fig. 3b,b_1_). Since the condition in which FAA is investigated during the dark phase of a LD condition is missing in our study, we cannot conclude whether the FAA deficit is independent of light.

### Seconds timescale

#### Time perception: *Afh* mutants exhibit delayed timing compared to wild-type mice

**Figure 4:**
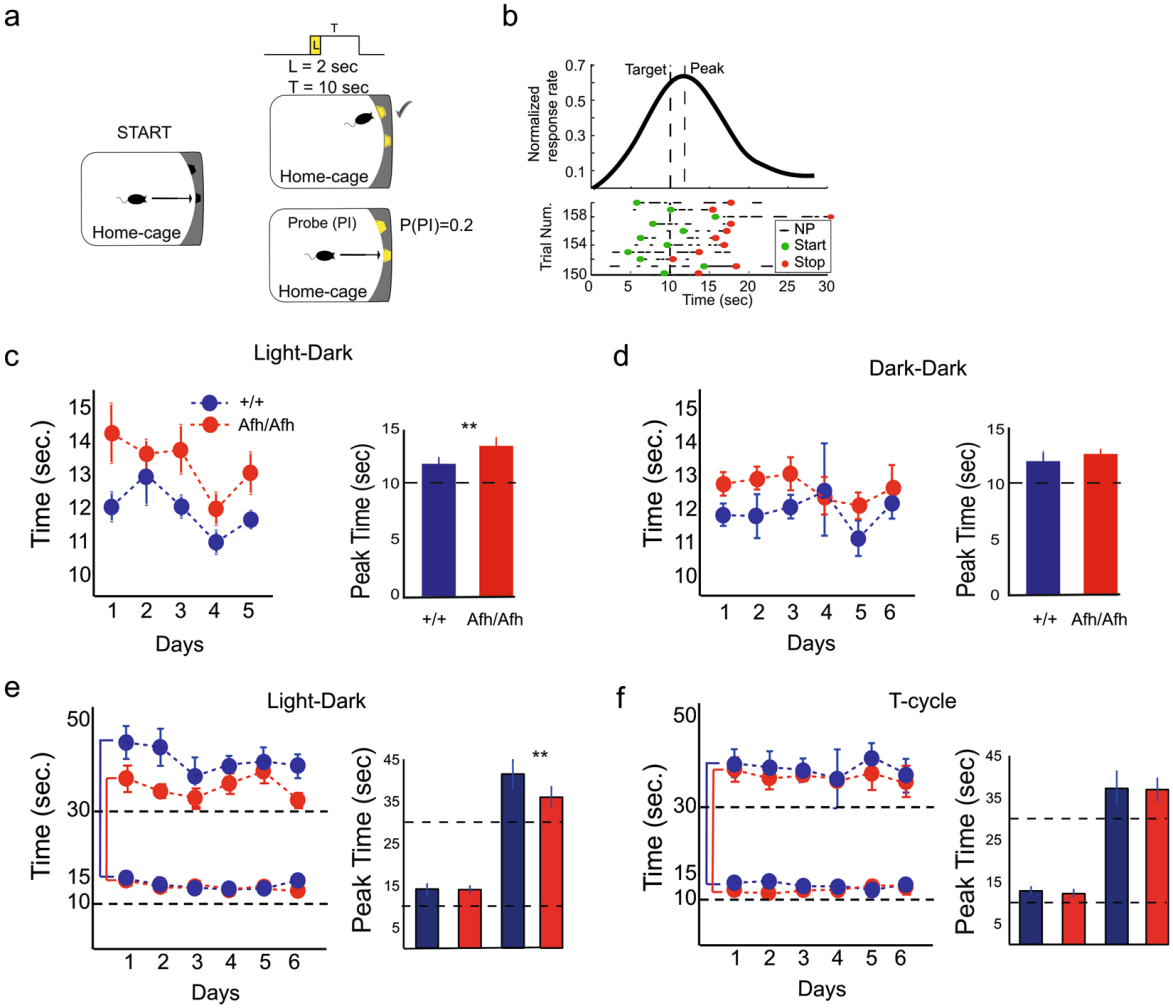
Interval Timing is disrupted in *Afh* mutants. (**a**) Graphical representation of the fixed-interval and peak-interval task. A nose poke in the central hopper activates a light signal (2 sec) and after 10 sec. the mouse receives a food pellet at the lateral hopper if it nose pokes before a time limit of 30 sec. In probe (PI) trials no pellet is dispensed. Probe (PI) trials are presented with a probability of 0.2. (**b**) Representative response curve gathered from probe trials. The increase of nose pokes around the target time reflects the trial time at which the animal’s expectancy of the reward delivery is maximal. The Peak times (mean ± SEM) in the timing task are shown for wild-type (n = 6) mice (blue) and for *Afh* (n = 6) mutants (red) over 5 consecutive days (left panel) and overall (right panel) in LD (**c**) and DD (**d**). Peak times (mean ± SEM) for the two-interval experiment are plotted over 6 days (left panel) and overall (right panel) in LD (**e**) and in the T-cycle (**f**) condition.

We studied time perception in *Afh* mutants and littermate control mice by testing them in the Fixed Interval and Peak Interval task in their work-for-food home-cage. The task involved collecting food pellets after a fixed delay (10 sec.) since the initiation of the trial (see Methods). In order to capture the interval timing behavior of mice without contaminating their timed anticipatory responding with reward delivery, reinforcement was omitted on average in one out of five trials (i.e., probe trials; Fig. 4a). The distance between the peak of the average response curve and the target interval (10 s; Fig. 4b) was used as an index of temporal accuracy. Our analysis revealed a significant delay in the peak times of *Afh* mutants compared to wild-type mice during the LD condition. This result held for each day of 5 day long testing (Fig. 4c). This finding suggests a remarkable similarity between the lengthening of the circadian clock in mutants and their differential processing of seconds-long time intervals. In both cases, the observed findings can be accounted for by assuming a decelerated clock. Surprisingly, the peak times of the *Afh* mutants were similar to those of wild-types in the DD conditions; this is shown by similar peak times per day between the two groups (Fig. 4d).

#### Time perception: *Afh* mutants fail to independently time short stimuli

Several conditions such as Parkinson’s disease (PD) are characterized by disruption in timing in two forms. PD patients over-reproduce a single interval whereas they over-reproduce the short interval and under-reproduce the long interval (i.e., migration effect) when these two intervals are presented in the same session^25^. Provided that we have observed over-reproduction of a single interval in *Afh* mutants and in order to test if a PD-like timing disruption is present in analogous conditions to the previous human work, we tested a different cohort of mice in another timing task. In this task, mice discriminated two durations differentially associated with two different lateral hoppers (10 s vs. 30 s; see Two Time Interval Task in Methods). Here, we observed a migration-like timing phenotype in *Afh* mutants in the LD condition when their performance was compared with the WT controls. *Afh* mutants over-estimated (under-reproduced) the timing for the longer stimulus (i.e., 30 sec.) in LD condition (Fig. 4e), suggesting that mutants fail to independently process two intervals similar to PD-related timing disruption in these conditions. Interestingly, the timing of the second duration restored during the lengthening of the T-cycle and the timing responses for the two independent targets were similar between the two groups (Fig. 4f).

### Decision-making

#### *Afh* mutant mice have a higher timing uncertainty compared to wild-type mice

**Figure 5:**
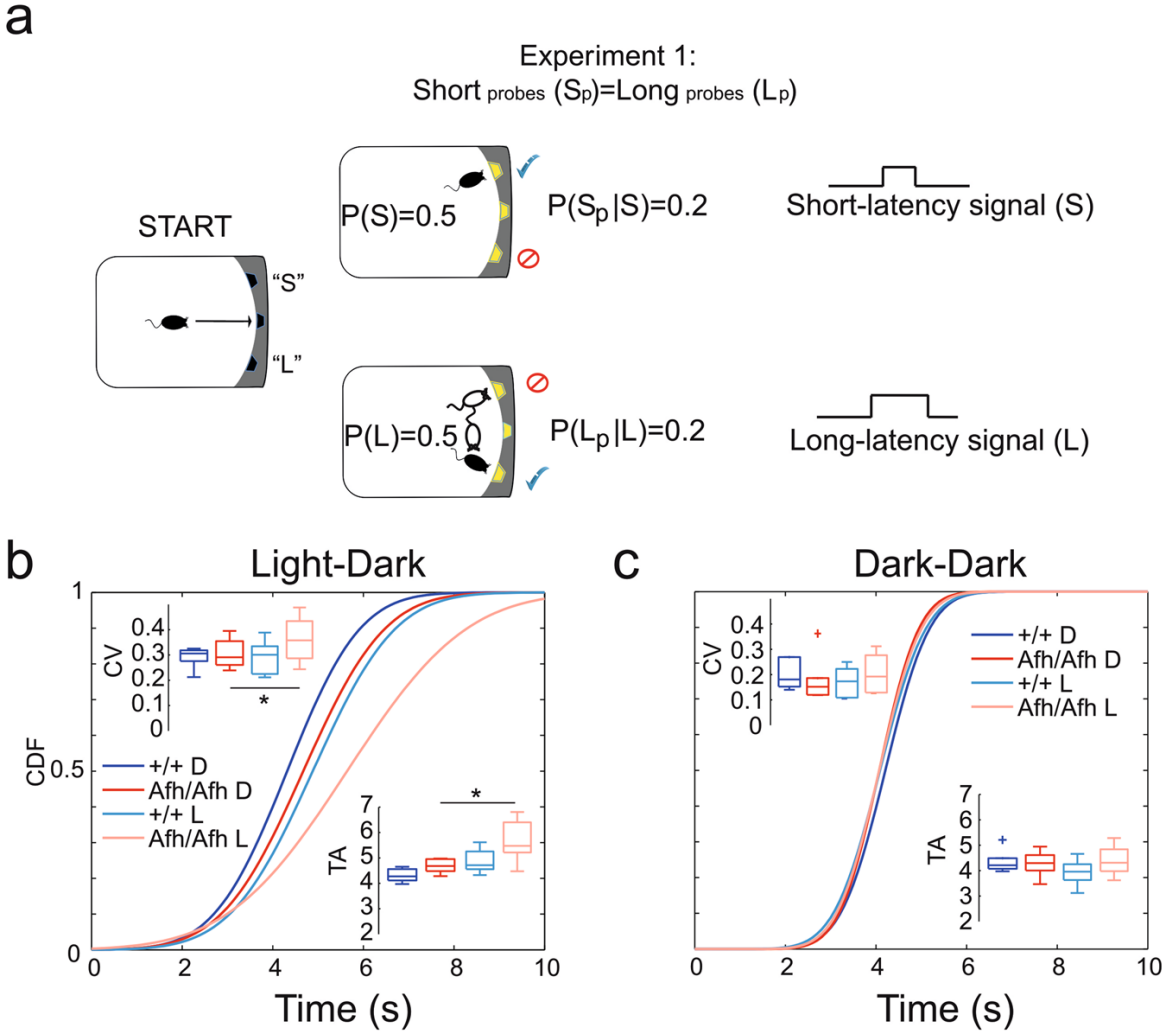
*Afh* mutants show higher timing uncertainty compared to control mice. (**a**) Representation of the Switch task in Experiment 1. Pellet-rewards are associated with two different lateral hoppers, based on the duration of the signal (e.g., short-signal (S) for left hopper and long-signals (L) for right hopper, counterbalanced). 20% of short- (S) and long- (L) signal trials were not rewarded (Probe trials, S_p_ and L_p_, respectively). All cumulative distribution functions, CDFs, of switch latencies are plotted for wild-type (n = 6) and *Afh* mutants (n = 6) for the dark phase (blue and red, respectively) and light phase (light blue and light red, respectively) in LD (**b**) and DD (**c**). The boxplots of the coefficient of variations (CV) and timing accuracy (TA) of switch latencies are plotted above and below the curves, respectively. In DD condition, values are computed for the subjective night (D) and subjective day (L).

Understanding temporal regularities associated with different options is critical for making adaptive decisions and assessing the risks associated with the corresponding behavioral responses. In order to address this function, we subjected groups of *Afh* homozygous mice and their wild-type littermate controls to a temporal decision-making task^13^, in which mice were trained to decide whether the stimulus was a short (S) or a long (L) signal in order to correctly predict the location of food delivery in the home-cage^16,23^. In this new test the pellet was dispensed contingent upon the response at the correct location (i.e., hopper) and correct time (see Switch Task in Methods). A mouse typically learns to initially nose-poke at the hopper associated with the short delay and if responding there is not reinforced after the short delay, it switches to the location associated with the long delay (Fig. 5a). In order to receive reward in the L trials, the switching from S-location to the L-location needs to occur before the end of the signal and adaptively deciding when to switch between hoppers requires risk assessment.

In this task, the main focus is the latency (trial time) at which mouse switches from S-location to L-location, which is conceptualized as temporal decision output (an accuracy measure). The coefficient of variation (CV) of switch latencies was used as a measure of timing uncertainty. The switch latencies were delayed (i.e., shifted rightward) in *Afh* mutants compared to wild-type control; in particular mutants showed significant delay in their timed decisions as well as higher CVs during light phase compared to the dark phase in LD condition (Fig. 5b), suggesting a higher timing uncertainty in mutant mice compared to wild-type mice. Once again, differences between the two groups disappeared in DD (Fig. 5c). To ensure that our results were related to timing and not to task performance differences we checked that the learning rate and food intake was similar between genotypes (Supplementary Fig. S1).

#### *Afh* mutant mice do not adjust temporal decisions in response to task parameters

**Figure 6:**
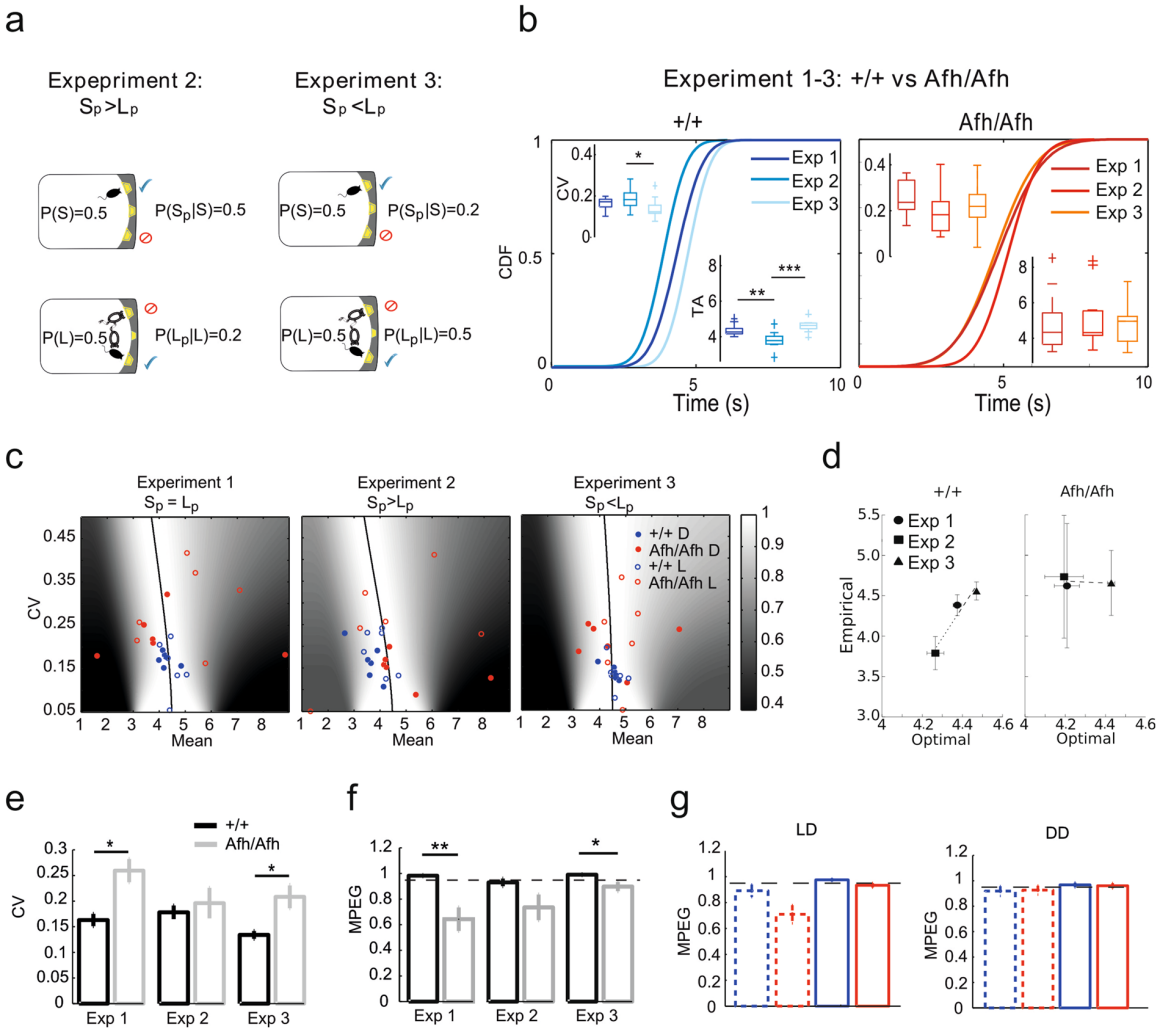
Probabilities and optimal decision-making: *Afh* mutants do not exhibit behavioral flexibility and make suboptimal decisions. Graphical representation of different experiments (Experiment 2 and 3) during switch task in which the probabilities of obtaining probe trials changed at short (S_p_) and long (L_p_) locations (**a**). Experiment 1 is shown in Fig. 5a. Comparison between the CDFs of the switch latencies during the three phases for each genotype (n = 6 wild-types; n = 6 Afh homozygous mice). The boxplots of the timing uncertainty (i.e., CV) and timing accuracy (TA) are shown above and below the cumulative curves. (Paired t-test within genotype, *p < 0.05, **p < 0.005, ***p < 0.001) (**b**). The three panels represent the expected gain surface for the three experimental conditions and the associated optimal performance curves (black solid line). We then plotted over the expected gain surfaces the empirical mean switch latency of each mouse separating them by their genotypes, in dark (filled circle) and in light (empty circle) phase (**c**). The empirical switch latencies as a function of optimal latencies in wild-type mice (left panel) and *Afh* mutants (right panel) for three experimental conditions (**d**). Timing uncertainty (i.e., CV) for each genotype and each experimental phase (**e**). Proportion of maximum possible expected gain (MPEG) in the three experimental conditions (**f**) as well as in LD (left panel) and DD (right panel) for Experiment 1 (**g**). Homozygous mice never reached the optimality (black dashed lines; values >0.95; *p < 0.05, **p < 0.005, One-way ANOVA). MPEG values are plotted per genotype (i.e., grey or red = *Afh*; black or blue = control mice) and phase (light/subjective day are the dashed lines; dark/subjective night solid lines).

To elucidate the ability of mice to modulate their temporal decisions in response to changing environmental statistics, we tested mice in the switch task with two different probabilities of probe (unrewarded) trials for different trial types (i.e., short vs. long; Fig. 6a). In order to avoid the biasing effect of data censoring in short-signal trials (due to the procedure), we analyzed the timed responses only in long-signal trials. The long-signal trials were characterized by equal (S_p_ = L_p,_ Experiment 1 discussed in the previous paragraph), higher (S_p_ > L_p,_ Experiment 2) or lower (S_p_ < L_p,_ Experiment 3) probability of obtaining rewards at long compared to short location.

In wild-type mice, we observed that a lower probability of obtaining a pellet in the short location (high probe trial probability, P(S_p_|S) = 0.5 vs P(L_p_|L) = 0.2) resulted in significantly earlier switch latencies whereas a higher probability of receiving a pellet in short location (low probe trial probability, P(S_p_|S) = 0.2 vs P(L_p_|L) = 0.5) compared to long location was associated with significantly delayed switch latencies (Fig. 6b, left panel). This modulation was predicted by reward maximizing strategies in these conditions^13^ and indicates that our wild-type mice modulated their temporal decisions according to the variation of external factors (i.e., probability of receiving reward at different locations). In contrast with the wild-type mice, *Afh* mutants did not modulate their switch latencies in response to different probabilities of receiving reward at different locations (Fig. 6b, right panel).

In order to further explore the adaptiveness of different decision strategies in response to changing task conditions, we compared the performance of wild-type and mutant mice in relation to the optimal decisions computed for each mouse. While the empirical switch latencies of control mice tracked the optimal latencies, the switch latencies of the *Afh* mutants did not (Fig. 6c). The empirical latencies correlated with the optimal performance curve in wild-type mice but not in *Afh* homozygous mutants (Fig. 6d). *Afh* mice had a significant higher CV (Fig. 6e) and a reduced proportion of maximum possible expected gains (MPEG) compared to wild-type mice (Fig. 6f). Moreover, consistent with some of our earlier results (Fig. 5c), no differences were observed in terms of proportion of MPEGs between the two groups in DD (Fig. 6g, right panel) compared to LD (Fig. 6g, left panel) condition.

### Sleep

#### Sleep homeostasis is altered in *Afh* mutants

In order to assess how *Afh* mutation affected the physiological aspects of sleep, we recorded electroencephalogram (EEG) and electromyogram (EMG) from *Afh* homozygous mice and their wild-type littermate controls for 48 h baseline (BS) under LD 12:12 standard illumination. Next, we tested the homeostatic response to sleep loss by subjecting all mice to 6 hours of sleep deprivation (SD), starting at zeitgeber time (ZT) 0, and then letting recovery for 42 consecutive hours. We scored all records for the three major states: Wakefulness (W), Rapid Eye Movement (REM) and Non-REM (NREM) sleep. We then analyzed the distribution of the three major states and the main EEG frequencies to derive the characteristics of their sleep. Wakefulness was confirmed in all sleep-deprived animals for the entire duration of SD.

**Figure 7:**
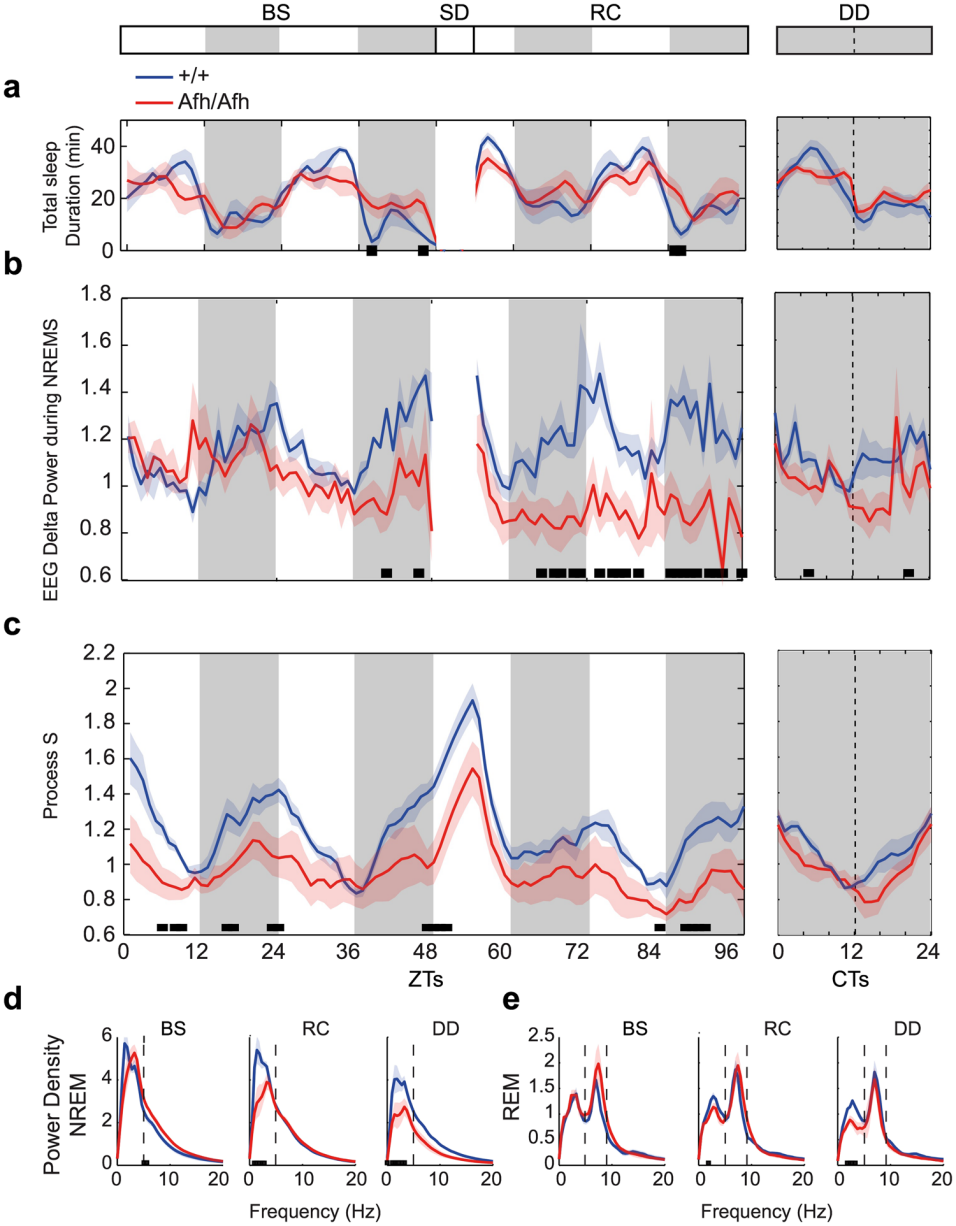
*Afh* mutants show disrupted sleep homeostasis. (**a**) Total sleep time in baseline (BS) and recovery (RC) period after sleep deprivation (SD) and in constant darkness (DD). (**b**) EEG delta power during NREM epochs is plotted for all phases. (**c**) Process S across all phases. Power density values for the entire BS, RC and DD phases are presented for NREM (**d**) and REM (**e**) epochs. All graphs are presented as mean ± SEM over either 1 hour intervals (**a–c**) or as frequencies (**d,e**). The black bars at the bottom of each graph indicate intervals where significant genotype differences were identified (n = 6 wild-types; n = 6 Afh mutants; P < 0.05, One-way ANOVA). The gray shadowed regions represent the dark phases. All graphs in DD are presented as CTs where data are rescaled according to subjective periodicities (see Methods).

During baseline, there were only minor sporadic differences in total sleep time between *Afh* homozygous mutants and wild-types (Fig. 7a, Table 1). A detailed analysis of the fragmentation of sleep revealed that *Afh* mutants presented a significantly higher number of NREM episodes compared to wild-type mice mainly in the dark phase (Supplementary Fig. S2a, Table 1), while the number of REM episodes was similar between the two genotypes (Supplementary Fig. S2b, Table 1).

**Table 1:** Summary of the statistical genotype differences for each phase of various sleep measurements (light/dark subjective day/subjective night - Repeated measure ANOVA: n.s.

Genotype Effect	Baseline				Sleep Deprivation (SD)	Recovery				Dark-Dark (DD)	
	Light	Dark	Light	Dark		Light (6 h)	Dark	Light	Dark	Subj. day	Subj. night
Tot sleep duration (min)	n.s.	n.s.	*	*		n.s.	n.s.	n.s.	*	n.s.	n.s.
NREM episodes (num)	n.s.	**	*	***		n.s.	n.s.	n.s.	*	n.s.	*
REM episodes (num)	n.s.	*	n.s.	n.s.		n.s.	n.s.	n.s.	n.s	n.s.	n.s.
EEG Delta Power during NREM	*	n.s.	*	***		*	***	***	***	*	***
Process S	***	***	**	***	**	*	***	***	***	*	**

not significant, *p < 0.05, **p < 0.005, ***p < 0.001).

Moreover, *Afh* mutants showed a reduced rebound of NREM delta power (Table 2) following sleep deprivation, indicating a reduced homeostatic response to acute sleep loss. NREM EEG delta power of *Afh* mutants was significantly reduced in baseline conditions and was not aligned with the light-dark cycle; it remained low over the entire recovery period (Fig. 7b, Table 1). Thus, we looked at the sleep process (Process S) as it was previously modeled for mice in literature^26^. This model combines the delta power values with the distribution of sleep-wake epochs to describe daily variations in sleep need (see Methods). Process S was significantly different between the genotypes over the entire recording period (Fig. 7c, Table 1), confirming a chronic sleep defect in *Afh* mutants. Although sleep pressure increased during the dark phase and decreased during the light phase for both genotypes, the major difference between *Afh* mutants and wild-type controls emerged at the end of the dark phase (Fig. 7c), when the sleep pressure for mice was maximum.

**Table 2:** Mean ± SEM of NREM and REM are reported in baseline (ZT-7) and after sleep deprivation (Post SD, at ZT-7) as well as the difference between the two values.

	baseline	Post-SD	difference
**NREM (min)**			
+/+	238 ± 27	308 ± 22^#^	70 ± 23.3
*Afh*/*Afh*	241 ± 26	299 ± 25^#^	58 ± 30
**NREM Fragmentation (n. episodes)**			
+/+	8 ± 1	13 ± 2^#^	5.2 ± 2.1
*Afh*/*Afh*	12 ± 1*	12 ± 0.8	0.38 ± 1.9*
**NREM mean episodes duration (min)**			
+/+	2.7 ± 0.4	2.3 ± 0.3	−0.4 ± 0.3
*Afh/Afh*	1.7 ± 0.1*	2.1 ± 0.1^#^	0.38 ± 0.2*
**NREM Delta Power**			
+/+	1.04 ± 0.04	1.47 ± 0.06^#^	0.45 ± 0.02
*Afh/Afh*	0.99 ± 0.09	1.17 ± 0.1	0.2 ± 0.1
**REM (min)**			
+/+	37 ± 2.4	53 ± 5.1^#^	15.9 ± 4.8
*Afh/Afh*	35 ± 6.6	38 ± 4.9*	3.1 ± 7.5

^#^Indicates the significant difference between baseline and Post SD within genotype (Paired T-test), *significant difference between genotype (Kolmogorov-Smirnov test).

Quantitative analyses of EEG frequencies during baseline, recovery and DD conditions revealed power density differences in the delta (1–5 Hz) frequency range but not in the theta (5–9 Hz) range (Fig. 7d) confirming that mutants suffered from a NREM deficit. During recovery from sleep loss, *Afh* mutants exhibited a significant reduction of the power density in the delta band for NREMs. Consistent with these latter results, only residual low frequency peaks in REMs, residing within the delta range, were different between the genotypes (Fig. 7e).

Overall, the sleep changes that occurred in the LD condition were attenuated in DD, but they were not completely eliminated; during constant darkness the total sleep time, sleep fragmentation, and delta power were similar between genotypes while the power density of delta frequency observed in DD remained reduced in *Afh* mutants.

### Molecular correlates

#### A pan-regional effect of the *Afh* mutation on the regulation of *Per* and *Cry*

**Figure 8:**
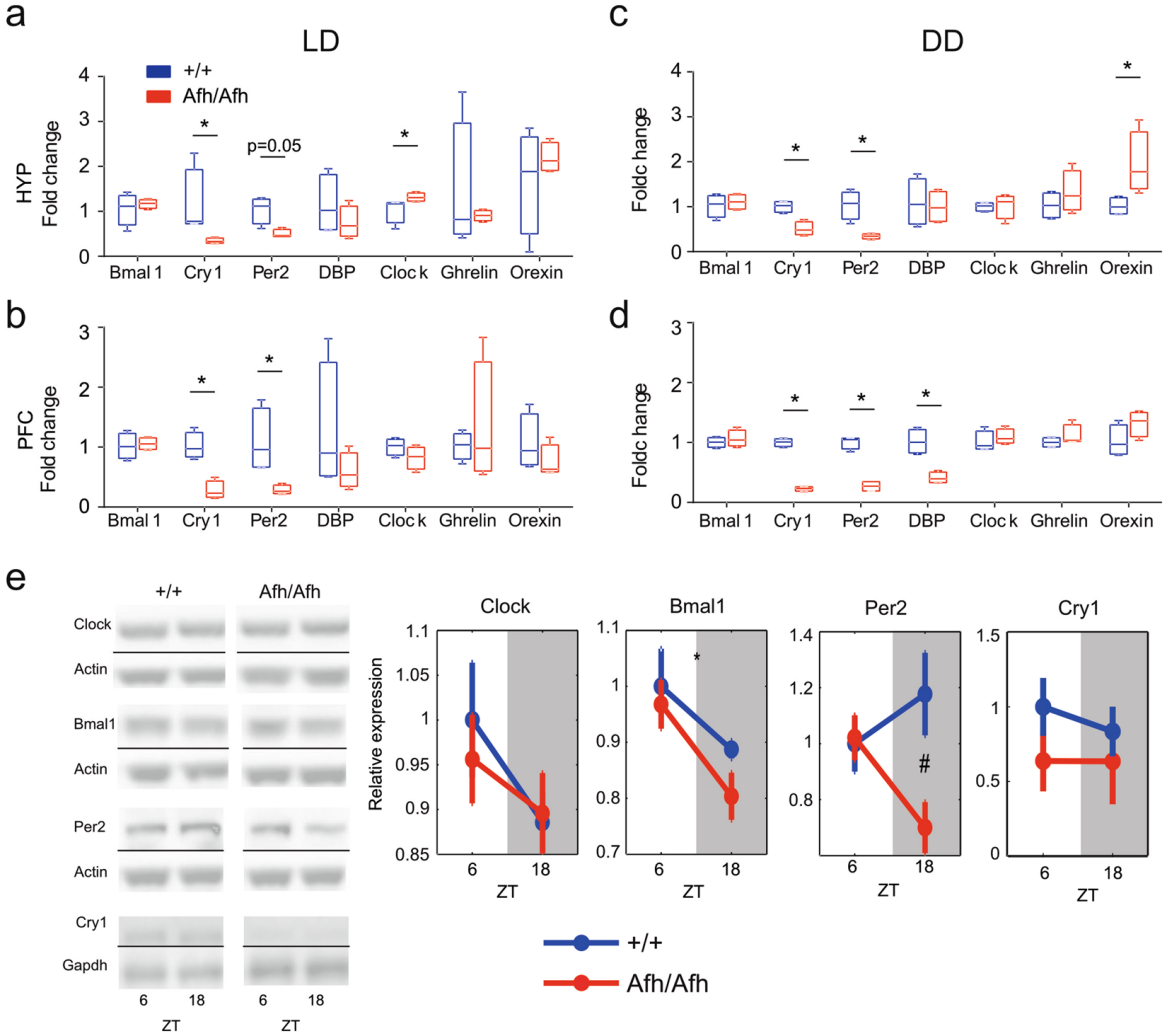
Clock molecular targets are abnormally expressed in *Afh* mutants in brain areas outside the SCN. Gene expression of clock and clock-related genes plotted for HYP and PFC in LD (**a,b**) and in DD (**c,d**). The asterisks above the graph indicate significant genotype differences (n = 4 wild-types; n = 4 *Afh* mutants; P < 0.05, One-way ANOVA). (**e**) Clock proteins in Striatum. Levels of clock proteins are shown at ZT6 and ZT18. Grey shadowed regions represent the dark phase (right panels). The (*) mark indicates significant effects of time while the (#) mark indicates significant genotype effects (n = 8 wild-types; n = 8 *Afh* mutants).

All of the abnormalities that *Afh* mutants expressed in the modulation of their daily activity, as well as the specific defects in interval timing and sleep, indicate that specific clock regulatory mechanisms may arise outside the SCN in these mutants. To investigate the molecular correlates of these behaviors, we conducted a gene expression profiling in hypothalamus (HYP) and pre-frontal cortex (PFC) collected in LD and DD in the food restriction experiments (see Gene expression analysis by quantitative real-time PCR in Methods). *Afh* mutants showed a significant reduction in the expression of *Per2* and *Cry1* in both hypothalamus (Fig. 8a) and pre-frontal cortex (Fig. 8b) in the LD. The same trend was also observed in DD condition (Fig. 8c,d). The reduced expression in *Per* and *Cry* was accompanied by an increase of *Clock* expression in hypothalamus, indicating that an alteration of *Per*/*Cry*-mediated transcriptional repression affects the Clock-mediated positive feedback loop. Furthermore, in constant darkness a hypothalamic increase of *Orexin* transcript (Fig. 8c) suggests that sleep-wake and food-anticipatory regulatory alterations in *Afh* mutants may originate from hypothalamic circuits. *Orexin* is a neuropeptide secreted in the hypothalamus that exerts a pivotal role in both sleep-wake regulation and food intake^27^. Moreover, we observed an increase of the albumin D-binding protein, *Dbp* (Fig. 8d) in the prefrontal cortex of wild-type mice in DD condition compared to *Afh* mutants. Dbp is a gene that is under the transcriptional control of CLOCK/BMAL and it is considered to have an important role in sleep homeostasis in mice^2^. These results confirm that the *Afh* mutation has a significant role outside the SCN, influencing local gene-networks according to region-specific biological functions.

#### Per2 is abnormally regulated in the striatum of *Afh* mutants

We complemented our investigation with the exploration of core clock proteins in striatum, the fundamental brain structure implicated for timing in the seconds-to-minutes range^12^. In order to study the circadian-dependent variations of proteins, we have collected tissues at ZT6 and at ZT18 of different cohorts of mice left under regular LD condition. As expected, all clock proteins were expressed in a circadian fashion (differing according to zeitgeber time) (Fig. 8e). For example, at ZT18 *Clock* and *Bmal1* levels were lower than at ZT6 while Per2 levels were higher in wild-type mice. Importantly, however, Per2 levels were reversed in *Afh* mutants, showing a significant genotype difference at ZT18 (Fig. 8e).

#### Monoamines in *Afh* mutants compared to controls

HPLC analysis showed that the levels of monoamines and of their metabolites in striatum changes according to the light phase. In particular, DA and its predominantly extracellular metabolite homovanillic acid (HVA) levels as well as 5HT and its metabolite 5-hydroxyindoleacetic acid (5HIAA) levels significantly change between light and dark, in both genotypes (Supplementary Fig. S3). Intraneuronal metabolite 3,4-dihydroxyphenylacetic acid (DOPAC) levels did not change significantly between light and dark phases but present with difference between +/+ and *Afh*/*Afh* animals (Supplementary Fig S3). DOPAC is an intracellular metabolite of DA that is known to be affected by changes in either DA synthesis^28^ or DA storage^29^ suggesting that one or both of these processes may potentially be altered in mutants. However, the limitation of our current study stands in the age difference of samples between the behavioral and the biochemistry study (see Methods).

### Circadian vs. sleep

#### Circadian clock and sleep differentially affect daily behaviors in mice

**Figure 9:**
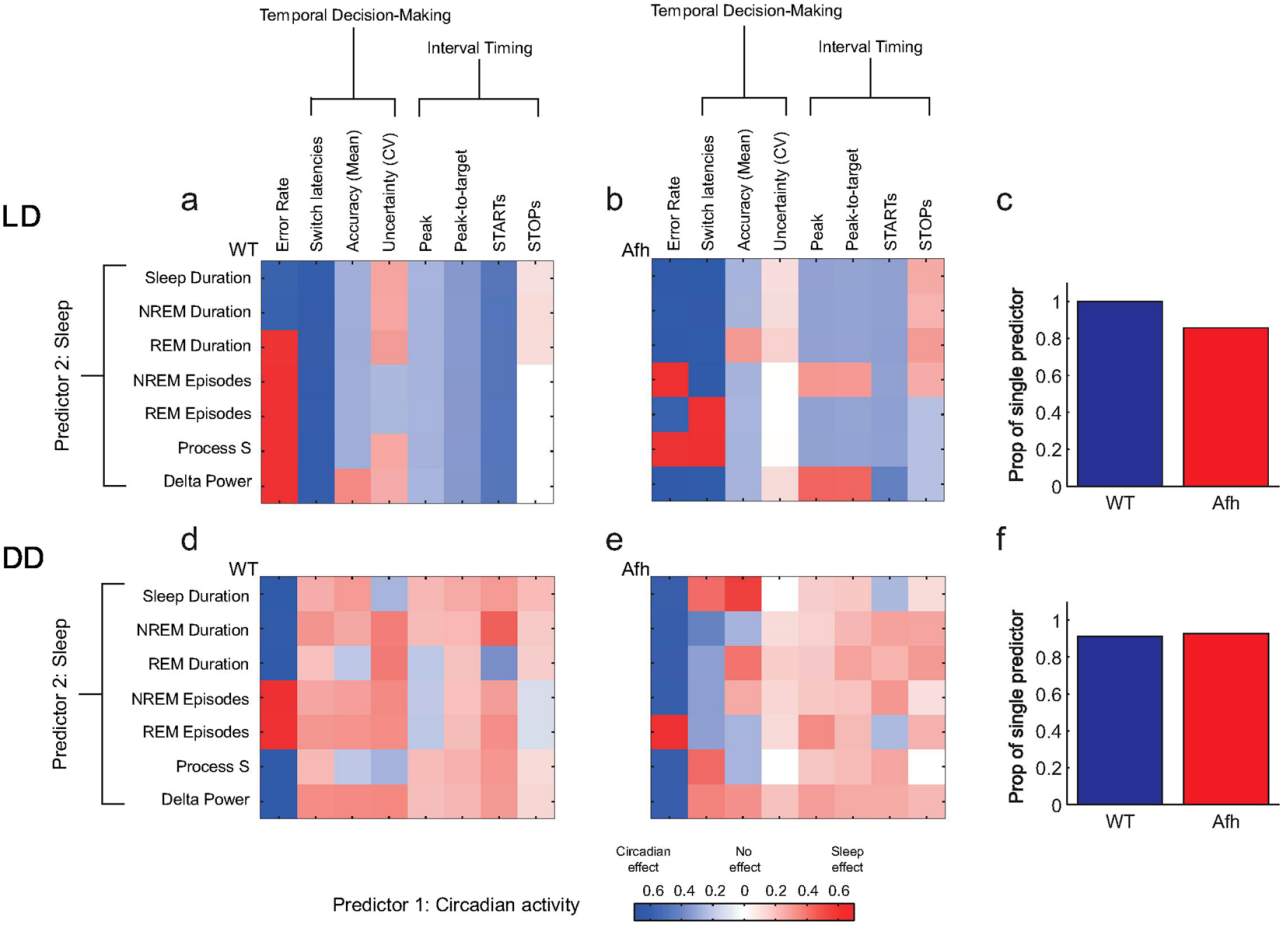
Multiple regression analysis (MRA) for circadian and sleep predictors over behavioral measures. MRAs are plotted for multiple behaviors in LD (upper panels, (a,b)) and DD (lower panels, (d,e)) and for wild-type (left panels, (a,d)) mice and *Afh* (right panels, (b,e)) mutants. Predictor 1 (circadian nose poking activity) and different sleep measures as predictor 2 were used. The color intensity reflects the R^2^ value of MRA corresponding to the best lag condition. The proportion of MRAs in which a single regressor was needed to predict the behavior in LD (**c**) and DD (**f**) condition.

We explored whether circadian and sleep information from our two groups of mice had any reliable relationship with the behavioral measures. To this end, we performed stepwise multiple regression analyses (MRA) based on “best lags” derived from linear regressions of behavioral observations on circadian or sleep predictors (see Methods). In Fig. 9 we displayed the output of the MRAs across the main behavioral parameters using two predictors (Circadian activity and one Sleep measure). The comparison between LD and DD in both groups showed a major contribution of the circadian predictor in LD (majority of blue squares, Fig. 9a,b), as result of the light-dependent oscillators, whereas the sleep predictor played a more meaningful role in DD (majority of red squares, Fig. 9d,e), in absence of external light cues. Moreover, the behavioral parameters were exclusively predicted by only one of the predictors (Circadian or Sleep, Fig. 9c,f) both in LD and DD conditions. This analysis predicted that circadian and sleep processes might play different roles in conditioning/modulating behavioral measures. We could identify how much of the variation in each behavior and for each genotype may be explained by each predictor. Interestingly, we observed that in many cases the best prediction (quantified by R^2^, see Supplementary Fig. S4) of the behavioral measures was influenced by circadian or sleep process differentially depending on the lag. In other words, the circadian clock system was a good predictor of behavioral outcomes at particular temporal lags, whilst the sleep process was a better predictor at other lags.

Moreover, our analysis revealed that the phase shift between the two predictors was significant (Supplementary Fig. S4). In wild-type mice the sleep predictor for behavioral observations such as error rate was in anti-phase with respect to the circadian predictor (Supplementary Fig. S4), peaking when the other presents a trough and vice versa. This effect was clearly demonstrated by cross-correlograms between the predictors, showing the trough of the curve at 0 lag in both LD and DD for wild-type mice (Supplementary Fig. S4c,d). Importantly, the interval between the trough and the peak of the curve indicates the hour lags that are necessary for the two predictors to be in phase. For *Afh* homozygous mutants the two processes were not in perfect antiphase in LD, however this mismatch was recovered in DD.

## Discussion

The results of this work shows that *Afh* mutant mice are characterized by a significant deficit in adjusting their behaviors in response to the temporally changing environment, which suggests a reduced temporal phenotypic plasticity. Importantly, this effect was manifested in mutants at multiple timescales. The investigation of the length of daily activity revealed a significant deficit in terms of the offset of the activity in *Afh* mutants. This is in agreement with the alteration of the interaction between *Per* and *Cry* in our mutants. In particular, the original ‘dual oscillator model’ specifically predicted that *Per1*/*Cry1* and *Per2*/*Cry2* are regulators of the morning (dawn) and evening (dusk) oscillators, respectively. Among the studies that attempted to independently validate single genes with single oscillators, the full model was disproved^3^; however, the strongest evidence that emerged in literature confirmed the role of *Per2* in α compression^3^. *Per2* has a critical role in phase delay, which is thought to result from posttranslational processes that affect the degradation of PER2 proteins^7^. Thus, our observation of *Per2* reduction in different brain areas outside the SCN is consistent with these conclusions. In particular, the alteration of *Per2* in hypothalamus, pre-frontal cortex and striatum during the dark phase of *Afh* mutants could explain the deficits that mutants showed in FAA, NREM sleep and interval timing, as all these phenotypes are associated with *Per2* functioning and occurred mainly during the dark phase. However, a limitation of the current work is that Per2 levels were not described in constant darkness where some of the abnormal phenotypes we described were restored.

Through the analyses of food anticipatory behavior, we were able to conclude that *Afh* mutants did not develop an anticipatory behavior since they did not show the typical increase of behavioral activity before the time of the meal in DD. However, they showed a sudden behavioral response at the onset of the light phase in the LD protocol. This latter response suggested that *Afh* mutant mice were able to associate the moment of the meal with the light on (e.g., learning of the stimulus-response association), however, an alternative explanation of this response relates to the high sensitivity of *Afh* mice to light^22^. Nevertheless, we did not observe any abnormal light-dependent arousal activity in our EEG investigation.

We observed that short-time (seconds range) perception was also abnormal in *Afh* mutants. Although our observations could be explained by decelerated clock hypothesis in line with the observations with the circadian clock^19^, the predictions of this account was not confirmed when *Afh* mutants were tested in the temporal discrimination task (i.e., Fig. 4e). This latter phenomenon is predicted by striatal dysfunctions supported by the *Per2* defects we reported in the striatum.

The investigation of temporal decision-making revealed an interesting aspect linked to the effects of the *Afh* mutation in our mice. *Afh* mutants were unable to adjust their time-based decisions in response to the different probabilities of reward delivery after different delays. This lack of adaptability in interval timing behavior to exogenous factors echoes the observed failure of these mice in adapting their daily activity to changes in temporal parameters at the circadian level. This latter correspondence raises the question whether temporal phenotypic plasticity is a process that involves behavioral adaptation across multiple timescales.

Our study copes with specific behavioral phenotypes but do not cover all possible phenotypes that could influence those we investigated here. For example, further investigations to test visual sensitivity, spatial recognition and metabolism would further refine our understanding of the behavioral repertoire of the Afh mutants. In any case, we believe that the possible effects of some of these factors would be minimal. For example, response selection depends marginally on the spatial abilities in our tasks given the short distance between hoppers in home-cage. Yet, preliminary investigation of how *Afh* mice perform in spatial cognition revealed no alterations (G. Lassi, personal communication). Additional investigations are also required to dissect any possible influence of genetic background or age-related phenotypes to the regulation of sleep and circadian sleep^30^. In addition, the altered light sensitivity in *Afh* mice^22^ could potentially affect some of the behavioral responses of this mouse line, however the full understanding of this phenomenon need further investigation.

We also identified mutation-dependent sleep-related alterations. *Afh* mutants expressed important sleep physiological abnormalities in baseline and during recovery from the sleep loss. While *Afh* homozygous mutants’ wheel-running^19^ and nose poking activity can mostly entrain to a LD cycle, we show here that their EEG delta power did not. Furthermore, the simulation of Process S showed a reduction of the amplitude in *Afh* mutants in both LD and DD conditions. This latter effect results from NREM fragmentation (i.e., increased NREM episodes) and low delta power expressed by *Afh* mutants compared to wild-type mice.

Our mutation leads to changes in the dynamics of the sleep homeostasis. The rebound following sleep deprivation is reduced in *Afh* mutants, suggesting a physiological deficit in the sleep process. In *Afh* mutants we observed chronic alteration of the sleep physiology (i.e., NREM fragmentation and delta power reduction), confirmed by an abnormal Process S. The sleep changes in baseline were followed by a prolonged delta power decrease after sleep deprivation (Fig. 7b). However, the total sleep time was not affected in mutants. Moreover, shortly after entering DD, when mice were not under a controlled light-dark schedule, the differences in sleep between wild-types and mutants were attenuated, indicating that light and/or light-dark cycles may have a disrupting effect on the physiology of sleep in *Afh* mice.

Finally, we provided statistical predictions of how specific contributions of the circadian and sleep processes may account for behavioral changes in wild-type and mutant mice. This analysis revealed that both circadian and sleep processes are good predictors of interval timing behaviors and temporal decision-making, confirming our previous findings^15,16,23^. Finally, this study delineates, for the first time, the time-locked temporal dynamics between circadian clock and sleep in modulating the daily profile of short-interval behaviors. Moreover, the *Afh* mutation disturbs these predicted dynamics between circadian and sleep processes in LD, while DD restores normal antiphasic behavior between circadian and sleep processes.

## Methods

### Mice and Husbandry

The *Afh* (after hours) mice were derived at the Medical Research Council (MRC) Mammalian Genetics Unit (Harwell, UK) from an ENU mutagenesis program, using ENU mutagenized BALB/c males which were crossed to C3H/HeH females. The colony was subsequently bred in IIT and backcrossed for more than 10 generations to C57BL/6 J. All mice were genotyped as described in^19^. All experimental procedures were conducted with age-matched groups of littermate male wild-type mice. The total number of animals, wild-type and *Afh*, used in this study was 72. Wild-type and homozygous mutant males were group-housed in the experimental room a week before the experiments with food and water *ad libitum* under 12:12 light/dark cycle (lights on from 7:00 to 19:00).

All animal procedures were approved by our institutional animal committee (‘*Organismo preposto al benessere degli animali’*, OPBA, IIT, Genova) and by the ethical national committee in Italy, for IIT Genova. The study followed ARRIVE guidelines (http://www.nc3rs.org.uk/arrive-guidelines).

### Sleep Wireless Experiment

Six wild-type and 6 *Afh* homozygous mice were subjected to sleep investigation at 12–16 weeks of age. All mice were anesthetized with Ketamine/Xilazine, 90–150 K/7.5–16X intra-peritoneally and implanted with telemetry transmitters (Data Sciences, F20-EET, Gold system) for recording electroencephalography, electromyography and body temperature (°C) as described in^16^. Two weeks post-surgery recovery time ensured a full recovery of normal sleep. Then, we started recording all the physiological signals for 48 hours (sleep baseline), followed by 6-hours sleep deprivation, from 7:00 to 13:00 by gentle handling and brushing. The recovery period consisted of 42-hours and an additional 48 hours-long data were acquired during constant darkness.

### Working-for-food and Interval Timing Tasks

Mice were singly housed and maintained for several days in a novel home-cage (Cognition & Welfare, COWE) apparatus developed by TSE Systems (Germany) to monitor nose poke activity in mice. Each home-cage was equipped with three holes/hoppers on a metal wall. The central hole is the control hopper and the lateral hoppers are connected to two feeders that dispense 20 mg dustless precision pellets (BioServ, USA) upon a trigger signal from the software. All three hoppers are equipped with infrared beams and LEDs controllable by the experiment code. The software allows implementing various behavioral protocols by setting combinations of LED stimuli and pellet dispensing. We implemented a protocol of food restriction, a variant of the fixed Interval (FI) and peak interval (PI) task^31^, a two time interval and the switch task^13,32^. Each of these tasks was preceded by a training phases to associate the light/hopper with the food pellet. Between pre-training and training phase, a small reduction in the total food intake was observed; however, the weight of the animals was checked every few days to ensure that the body weight was stable and therefore not affecting other mechanisms such as metabolism. If an animal lost more than 15–20% of its body weight, it was removed from the cage.

#### Food Restriction

During this experiment, only two hoppers were available, the central and one lateral hopper. Mice were first trained to receive pellets from the lateral hopper (Pre-training phase). During this phase a central nose poke activated the light in the central and later hoppers for 2 seconds (L = 2 s). The mouse received a pellet reward if it was poking in the later hopper after the light signal and before the time limit (30 seconds). Then an inter-trial interval (ITI) started as a 30 s fixed interval plus a random delay drawn from a geometric distribution with mean of 60 s. The pre-training phase lasted for 3 days. Then a training phase started and pellets were only available between 10:00 and 16:00 either during the light phase of LD or in constant darkness. The trials could not be initiated and the nose pokes were not reinforced during any other period. This experiment was conducted with 6 wild-types and 6 *Afh* homozygous mice at 12–16 weeks of age. An LD phase of 14 days was followed by a 10 day-long DD phase. The analyses of LD and DD phases were limited to the last 10 and 7 days, respectively.

#### Fixed Interval and Peak Interval Task

A different cohort of mice was tested on the Fixed Interval (FI) and Peak Interval (PI) tasks. This experiment was conducted with 6 wild-types and 6 *Afh* homozygous mice at 12–16 weeks of age. Mice had access to food over the entire 24 hr and no food restriction was implemented.

#### Pre-training

The pre-training for the task aimed to train the mice to associate the light/hopper with food pellet. Mice self-initiated the trial by nose poking in the central hopper and this resulted in 2 sec illumination (L = 2 sec) of the two hoppers. If a mouse nose poked in the lateral hopper after the light signal and before the time limit (30 seconds), a pellet was delivered. The inter-trial interval (ITI) was defined as a 30 s fixed interval plus a random delay drawn from a geometric distribution with mean of 60 s. The pre-training phase lasted for 3 days.

#### Training

In this phase, we implemented the FI and PI tasks. In FI trials, the first response following the fixed interval (10 seconds) resulted in the delivery of the food pellet. The FI trials serve to train the mice with the reward availability time (i.e., target interval). However the reward delivery in FI trials contaminates responding and thus do not allow the characterization of the timing performance following the reward availability time. In order to capture the entire temporal expectancy of reward delivery, PI trials are introduced. The PI trials last longer than the FI trials and the reinforcement is omitted; the timing performance of the mice are evaluated based on response rates as a function of trial time (Fig. 4b).

Unlike in pre-training, in FI trials mice had to wait 10 sec (L = 10 sec) before receiving a pellet. In the training phase, 20% of trials were PI (probe) trials in which reinforcement was omitted. An LD phase of 7 days was followed by a 10 day-long DD phase. Only the data in PI trials from the last 5 days (steady-state) of LD phase and the last 6 days of DD were analyzed.

#### Two Time Interval Task

A different cohort of mice (6 wild-type and 6 *Afh* homozygous male mice at 12–16 weeks of age) was tested in the two time interval (dual peak interval) task. Mice had access to food for 24 hr. In this task, all three hoppers were available.

#### Pre-training

The pre-training phase aimed to train mice to form an association between light stimulus and reward. Mice self-initiated the trial with a nose-poke in the central hopper. This nose-poke activated the central light and one of the two lateral hopper lights that was randomly picked with equal probability (50%). The hopper was kept lid until the first nose-poke on the lateral hopper. Upon correct response, the light was switched off and a pellet was delivered, marking the end of the trial and the beginning of the ITI. The ITI was defined as for the other tasks. This phase lasted 3–4 days in LD condition (12:12).

#### Training

Two time interval task was implemented in this phase. Compared to the pre-training phase, here the short light signal of 10 sec (t_short_) was associated with the left hopper and the long light signal (t_long_ = 3*t_short_) of 30 sec was associated with the right hopper. Reward was available only in the FI trials. In order to receive a pellet, in FI trials the animals had to nose-poke in the lid hopper between the times of the light off and 3 times the light duration (10–30 sec interval for left hopper; 30–90 sec interval for right hopper). The reward was delivered in FI trials only if the first nose-poke after the light off was at the correct location, which initiated the ITI. After 3–4 days of training in this phase, the probe (PI) trials were introduced.

Probe trials occurred randomly with a probability of 0.2 for each side. During the probe trials the central nose-poke activated the light at the central and one of the lateral hoppers for a total duration of 90 sec. This phase lasted two weeks: one week in LD condition (12:12) and another week in T-cycle (LD 13.25:13.25). The probe trials were introduced in order to estimate whether interval timing behavior of the animals around the short (10 sec) and long (30 sec) target varied between different LD conditions. ITI started after 90 sec.

#### Switch Task

For this experiment, we tested a different cohort of mice at 12–16 weeks of age in the same home-cage system described above. During the pre-training phase, mice self-initiated the trial with a nose-poke in the central hopper. No temporal limitations were imposed during this phase and the trial ended when the animal poked and received a pellet on each side, determining the end of the trials and offset of the lights. Every trial was followed by an inter-trial interval (ITI). This phase lasted 3–4 days in LD condition (12:12).

All mice were trained in the switch task as described in^13,16,23^. The task required the discrimination of two light signal durations (i.e. short- vs. long-latency signals) in order to obtain a food pellet in a trial (Fig. 5a). The short (S) or long (L) duration of the light signal predicted the location of the pellet availability. Short and long trials were randomly intermixed with equal probability (P(S) = P(L) = 0.5). The first nose-poke (NP) in the correct location (left hopper after short signal and right hopper after long signal) was reinforced with one pellet. Incorrect responses, namely the nose-pokes in right hopper after short signal or nose-pokes in left hopper after long signal, terminated the trial without pellet delivery. The ratio between short and long signal was 1:3 (3 sec vs 9 sec) at the beginning and lasted for a week; it was then reduced to 1:2 (3 sec vs 6 sec) for few days.

After few days of switch task with 1:2 ratio, probe trials were introduced for both short-latency and long-latency trials. During probe trials the signal was presented as in regular trials but the responses of the animals were never reinforced, providing a partial reinforcement schedule for the mice.

The three experimental phases were differentiated by the probability of probe trials. In the first phase, short (S_p_) and long probes (L_p_) were introduced with the same conditional probabilities: P(S_p_|S) = P(L_p_|L) = 0.2 (Fig. 5a) whereas the second experimental phase contained the following unequal probabilities: P(S_p_|S) = 0.5 and P(L_p_|L) = 0.2 (Fig. 6a, left panel). These probabilities were reversed for the third phase: P(S_p_|S) = 0.2 and P(L_p_|L) = 0.5 (Fig. 6a, right panel). ITI started at the end of each trial. Each phase lasted one week.

Another cohort of 6 wild-type and 6 *Afh* homozygous mice (12–16 week old) were tested with 1:2 ratio after a pre-training phase in the LD condition for 11 days followed by the DD condition for 9 days. Only the last 5 days of the dataset were analyzed for LD and DD conditions, to ensure a steady state performance and entrainment with the new cycle (Supplementary Fig S1).

### Gene expression analysis by quantitative real-time PCR

Four Afh homozygous mutant mice and 4 littermate controls were sacrificed at ~7:30 for the LD protocol and at ~9:30 for the DD protocol at the end of the working-for-food cognitive task. Prefrontal cortex (PFC) and hypothalamus (HYP, excluding SCN) regions were dissected using a brain slicer matrix (Zivic Instruments, Pittsburgh PA USA) and they were immediately frozen in dry ice. Total RNA was extracted from approximately 0.5 g of snap frozen PFC and HYP using Qiazol (Qiagen, Hilden, Germany) according to the manufacturer’s instructions. RNA samples were quantified with an ND1000 Nanodrop spectrophotometer (Thermo Fisher Scientific Inc., Waltham, MA, USA). Reverse transcription of 0.5 μg of RNA was performed using ImProm-II(TM) Reverse Transcriptase (Promega, Milan, Italy) according to the manufacturer’s instructions. RT-qPCR was performed on an Applied biosystems 7900HT Fast Real-Time PCR System (Applied Biosystem, Foster City, CA) using QuantiFast SYBR Green PCR Kit (Qiagen, Hilden, Germany) and under the following conditions: 5 min at 95 °C, 40 cycles of denaturation at 95 °C for 10 sec, an annealing step at 60 °C for 30 sec and extension step at 70 °C for 1 min. Each sample was run to obtain average Ct values according to the manufacturer’s specifications. A list of the primers used is listed below. All samples were normalized against 2 different house-keeping genes: Gapdh and β-actin. Expression levels relative to these house-keeping genes were determined by the calculation of ΔCt. The data are expressed as 2- ΔΔCt, where ΔΔCt is the difference between the control and Afh mutant cohorts. In the case of Cry1 and Gapdh, the bands are from the same gel and the same exposure (Supplementary Fig. S5). For the other genes (Clock, Bmal1 and Per2) the membranes have been cut to detect several antigens for the same samples and the same parameters have been used to acquire the images.

The primers used are listed here:

*Bmal1* Forward CCGTGCTAAGGATGGCTGTT

*Bmal1* Reverse TTGGCTTGTAGTTTGCTTCT

*Clock* Forward TTGACAGAGATGACAGTAG

*Clock* Reverse TTACCAGGAAGCATAGAC

*Cry1* Forward GCTATGCTCCTGGAGAGAACGT

*Cry1* Reverse TGTCCCCGTGAGCATAGTGTAA

*Per2* Forward AGCTACACCACCCCTTACAAGCT

*Per2* Reverse GACACGGCAGAAAAAAGATTTCTC

*Dbp* Forward GAGCCTTCTGCAGGGAAACA

*Dbp* Reverse GCCTTGCGCTCCTTTTCC

*Grhelin* Forward CATGCTCTGGATGGACAT

*Grhelin* Reverse TGGTGGCTTCTTGGATTC

*Orexin* Forward CTCCAGGCACCATGAACTT

*Orexin* Reverse CAGTAGCAGCAGCAGCAG

*Gapdh* Forward GAACATCATCCCTGCATCCA

*Gapdh* Reverse CCAGTGAGCTTCCCGTTCA

*β-Actin* Forward AAGTGGTTACAGGAAGTCC

*β-Actin* Reverse ATAATTTACACAGAAGCAATGC

### Biochemistry

Eight wild-type and 8 *Afh* homozygous mouse were euthanized at 35–38 weeks of age by decapitation, at ZT6 and at ZT18. The right and left hemisphere striatum were rapidly dissected (within 60 sec) on an ice-cold surface and frozen in liquid nitrogen. One hemisphere striatum was used for Western blot analysis and the other for neurochemical measurement of monoamine tissue levels.

### Antibodies and Western blot Analysis

The anti-BMAL1, anti-CLOCK antibodies were purchased from Abcam and anti-Per2 from SantaCruz Biotechnology. Western blot analyses of brain samples were performed as described in^33^. Tissue samples were homogenized in boiling 1% SDS solution supplemented with protease inhibitor cocktail (Sigma) and boiled for 10 min. Protein concentrations were measured using a BCA-protein assay (Themo Scientific). Protein extracts (25 or 50 µg) were separated on 10% SDS/PAGE and transferred to nitrocellulose membranes. Blots were incubated with primary antibodies overnight at 4 °C. Immune complexes were detected using appropriate peroxidase-conjugated secondary antibodies (Jackson Immuno-Research) and a chemiluminescent reagent (Super-Signal West-Pico; Pierce Biotechnology). Densitometric analysis was performed within the linear range using IMAGEQUANT V1.1 (GE Healthcare Life Sciences). For quantitative analysis, actin was used as loading controls. The Western blot results were normalized to the respective control wildtype values at ZT6.

### Neurochemical measurement of monoamine tissue levels

The striatum was dissected rapidly on ice and frozen in liquid nitrogen. Tissue was homogenized in 40 volumes of 0.1 M HClO4, the homogenate was centrifuged at 10.000 × g for 10 min to remove debris. Subsequently, supernatants were filtered (Millipore Ultrafree-MC centrifugal filter units, 0.22 µm) and analyzed by HPLC as described below.

#### Analytical procedure

Measurements of DA and 5HTmetabolites in collected brain samples were performed by HPLC with electrochemical detection (ALEXYS LC-EC system, Antec Leyden BV, Netherlands) equipped with a reverse-phase column (3 μm particles, ALB-215 C18, 1 × 150 mm, Antec) at a flow rate of 200 μl/min and electrochemically detected by a 0.7 mm glass carbon electrode (Antec; VT-03). The mobile phase contained 50 mM H3PO4, 50 mM citric acid, 8 mM KCl, 0.1 mM EDTA, 400 mg/l octanesulfonic acid sodium salt and 10% (vol/vol) methanol, pH 3.9. The sensitivity of the method permitted detection of ~3 fmol DA.

## Data Analyses

### Sleep data analyses

EEG data recordings were analyzed with SleepSign software. A combination of automatic sleep scoring and manual correction was used. Scoring and extrapolation of the power spectra were performed on 4-second epochs. Each epoch was subjected to FFT (Fast Fourier Transformation) and classified as wakefulness (W), non-rapid-eye-movement (NREM) or rapid-eye-movement (REM).

The sleep scoring was analyzed in hourly bins and various parameters were extracted such as the total sleep time by combining the NREM and REM duration. The fragmentation was expressed as the number of episodes/hour, where episode is a period of one or more equal consecutive 4-seconds epochs. The EEG delta power during NREM epochs was expressed as the percentage of the average values of Delta Power during the last 4 hours of the light phases of the baseline^26^. The EEG delta power during NR epochs in DD was normalized by the average values of the last 4 hours of the subjective day (CT 7–11).

### Process S

The homeostatic process of sleep, Process S, has been mathematically reproduced in mice by Franken *et al*.^26^. The model recapitulates the increase and decrease in the need for sleep with two saturating exponential functions based on the sleep-wake distribution. In our study the parameters (asymptotes, time course, initial value) were estimated on the first day of the baseline for each subject. Process S was simulated during the SD and RC phases and was expressed as a percentage of the individual mean delta power over the last 4 hours of the light phase of the baseline during NREM epochs. Process S during the DD phase was rescaled according to the subjective period and normalized by the average values of the last 4 hours of the subjective day (CT 7–11).

A robust estimation of the subjective period was computed on the last 60 hours of continuous darkness of the Process S. We defined a matrix of cross-correlation values for the Process S, as described by equation (1):

1 $$Conv(\tau ,\delta )=P({S}_{24}(\delta ),{S}_{24}(\tau ))$$

where *P* is the Pearson’s correlation coefficient, *S*_24_ corresponds to a 24 hour long segment of the Process S taken at any δ and *τ* instants varying between 1 and L-24, where L corresponds to the length of the data set.

The cross-correlation of the Process S showed periodic diagonal structures that peaked in band regions of about 24–27 hours periodicity from the autocorrelation diagonal, where peaks reflected the intrinsic periodicity of the Process S. We refer to the maximum values of the *Conv*(*τ*, *δ*) in the diagonal structure corresponding to the bands τ = 0, δ ∈ [18, 30] and *τ* ∈ [18, 30], δ = 0 as *SecondPeak*. To robustly estimate the subjective period we first defined the weighting function for every *τ* and δ:

2 $$w(\tau ,\delta )=\{\begin{array}{c}Conv(\tau ,\delta ),\,\\ 0,\,\end{array}\,\begin{array}{c}when\,Conv(\tau ,\delta ) > 0.8\ast Second\,Peak\\ otherwise\end{array}$$

Then we defined the subjective period (*SP*) as the weighted average:

3 $$SP=\,\frac{{\sum }_{\tau ,\delta }w(\tau ,\delta )\cdot \tau }{{\sum }_{\tau ,\delta }w(\tau ,\delta )},\forall \tau {\epsilon }[1,L-24]$$

Finally, the onset of the subjective night for the Process S was defined as the time at which it reaches the maximum.

### Behavioral Data Analysis

#### Circadian Activity

Circadian periods of nose poking activity in mice were analyzed for each animal as described in Maggi *et al*.^23^. The analyses were performed using the MATLAB (www.mathworks.com) platform. The estimation of circadian period was computed with nonlinear curve-fitting of the nose pokes as a function of the recording hours with a sigmoidal function for various periodicities. A cross-correlation coefficient (CC) was calculated for each fit to evaluate the robustness of the nose-poke periodicity. The time devoted to nocturnal behavioral activity (alpha) was analyzed and computed with the algorithm described in Leise *et al*.^34,35^.

#### Peak Time in Peak Interval Task

Probe trials in the peak interval task were introduced to assess temporal accuracy and uncertainty of the mice. Data gathered from the probe trials can be expressed in two different ways. Averaging data across trials provides a nearly bell-curve shaped normalized response curve with its peak located at around the target duration. Data in our study were analyzed by modeling nose poking as a function of trial time at the single trial level as a three-state system (break → run → break). The two break periods are characterized by a relatively low rate of responding and the run period is characterized by a relatively high rate of responding^23^. The trial time at which the animal switches from low-to-high rate of responding was defined as the START time and the subsequent point at which the animal switches from high to low rate of responding was defined as the STOP time. These points were detected using an algorithm as described in^4,23^. The average of START and STOP times were used as the estimates of the Peak Time in individual trials (also referred to as middle time).

#### Temporal Decision-Making in the Switch Task

The primary unit of analysis in the Switch task was the trial time at which mouse left the short-latency location for the long-latency location in the long-signal trials (switch latency). The distributions of switch latencies were fit with a mixture of Gaussian distributions (parameters were chosen with a maximum likelihood estimation of the empirical data) as we have previously described^4,23^. The component of the distribution function with higher proportion (that explains the larger portion of the data) and with the estimated parameters within the range of interest (between short and long signal durations used in the task) was considered to represent the empirical distribution of the switch latency values.

The target-switch latency in this discrimination procedure was defined as the mean departure (or switch) latency and the endogenous timing uncertainty was quantified as the coefficient of variation (CV = standard deviation(data)/mean(data)), a measure of relative dispersion of the switch latency distribution^11^. The optimal target-switch latency was formulated taking into account the subjective timing uncertainty^13^ and the expected gain function used to this end was already generalized in our prior study to include the probe trial probability^11^. The Expected Gain (EG) function estimates, *a priori*, the gains associated with all possible switch latencies given the subjects’ endogenous timing uncertainty and other relevant task parameters such as the payoffs and probabilities.

For each subject, the optimal target switch latency was defined as the switch latency that maximized the EG given the subject’s CV and the task parameters. The ratio of the EG estimated for the empirical switch latency to the EG associated with the optimal switch latency is referred to as the proportion of maximum possible expected gain (MPEG). This value can vary between 0 and 1, where 1 is obtained when the mouse exhibits optimal switch latencies. The performance for each mouse was assessed by an index of error rate per hour, defined as E_i_/T_i_ where E_i_ is the number of incorrect trials (hour i = 1, ..., 24) and T_i_ is the total number of trials per hour.

### Multiple regression analysis with a stepwise method

To further assess the contribution of the circadian process (Predictor 1) and sleep processes (Predictor 2) to the modulation of all behavioral measures, we investigated which process was the best predictor of behavioral parameters (e.g. error rate, decision-making and interval timing parameters). Thus, we performed a multiple linear regression analysis, using a stepwise method. All the behavioral data and predictors were averaged within genotype and collected in one hour-long bins (24 ZT in LD condition, 24 CT in DD condition). We performed a linear regression of each individual behavioral parameter (i.e. error rate, switch latencies, etc.) on Predictor 1, defined as the Circadian activity (nose-poking activity) or on Predictor 2, defined as sleep parameters (i.e. sleep duration, NREM, REM, etc). Each regression was performed 24 times every time by shifting Predictor 1 or Predictor 2 forward by one time bin (i.e. lag) with respect to the behavioral parameter. For each model, we computed the R-squared statistics.

For each predictor we identified the “best lag” as the maximum R-squared achieved among the 24 regressions (i.e., 24 different lags). All data were reorganized and aligned with respect to its best lag. Then a multiple regression analysis conducted for each combination of Predictor 1 and Predictor 2 provided us with the best predictor for each behavioral parameter with its contribution (defined by the R-squared).

To highlight the phase relationship between the Predictor 1 (circadian) and Predictor 2 (Process S), we performed a cross-correlation analysis between the R-squared profiles of the two processes. The phase relationship was then evaluated based on the time lag of the cross-correlation peak.

### Statistical Analysis

Data were analyzed with one-way ANOVA for differences in a single time point. We used the Statistics Toolbox of the Matlab package for statistical analysis. The significant differences were indicated for p-values: *p < 0.05; **p < 0.005; ***p < 0.001. For each test we checked the F-statistic (which was always ≫ 1) and the statistical power, which depends on the sample size, the effect size (F-statistic) and significance level. All the tests that resulted to be significant had asymptotically high statistical power.

## Electronic supplementary material

Supplementary Figures
