# Supplementary material for: The after-hours circadian mutant has reduced phenotypic plasticity in behaviors at multiple timescales and in sleep homeostasis
Source: Sci Rep. 2017 Dec 19;7:17765. doi: 10.1038/s41598-017-18130-2 (PMC5736711; doi:10.1038/s41598-017-18130-2)
Supplement: Supplementary file 1 — Supplementary Figures [file 41598_2017_18130_MOESM1_ESM.pdf]

The after-hours circadian mutant has reduced phenotypic plasticity in behaviors at multiple timescales and in sleep homeostasis

Silvia Maggi[1,2], Edoardo Balzani[1], Glenda Lassi[1], Celina Garcia-Garcia[1], Andrea Plano[1], Stefano Espinoza[1,] Liudmila Mus[1], Federico Tinarelli[1], Patrick M. Nolan[3], Raul Gainetdinov[1], Fuat Balci[4], Thierry Nieus[1], and Valter Tucci[1*]

[1]Department of Neuroscience and Brain Technologies – Genetics and Epigenetics of Behaviour - Istituto Italiano di Tecnologia, via Morego, 30, 16163 Genova, Italy;

[2]Faculty of Biology, Medicine and Health, University of Manchester, Manchester, M13 9PL, UK

[3]MRC Harwell, Harwell Science and Innovation Campus, Oxfordshire, OX11 0RD, UK;

[4]Koç University, Department of Psychology, Istanbul, Turkey

[*]Correspondence should be addressed to Valter Tucci (valter.tucci@iit.it). Via Morego, 30, 16163 Genova, Italy; Tel. 0039 010 71781747. Fax: 0039 010 71781230.

- Supplementary Figures -

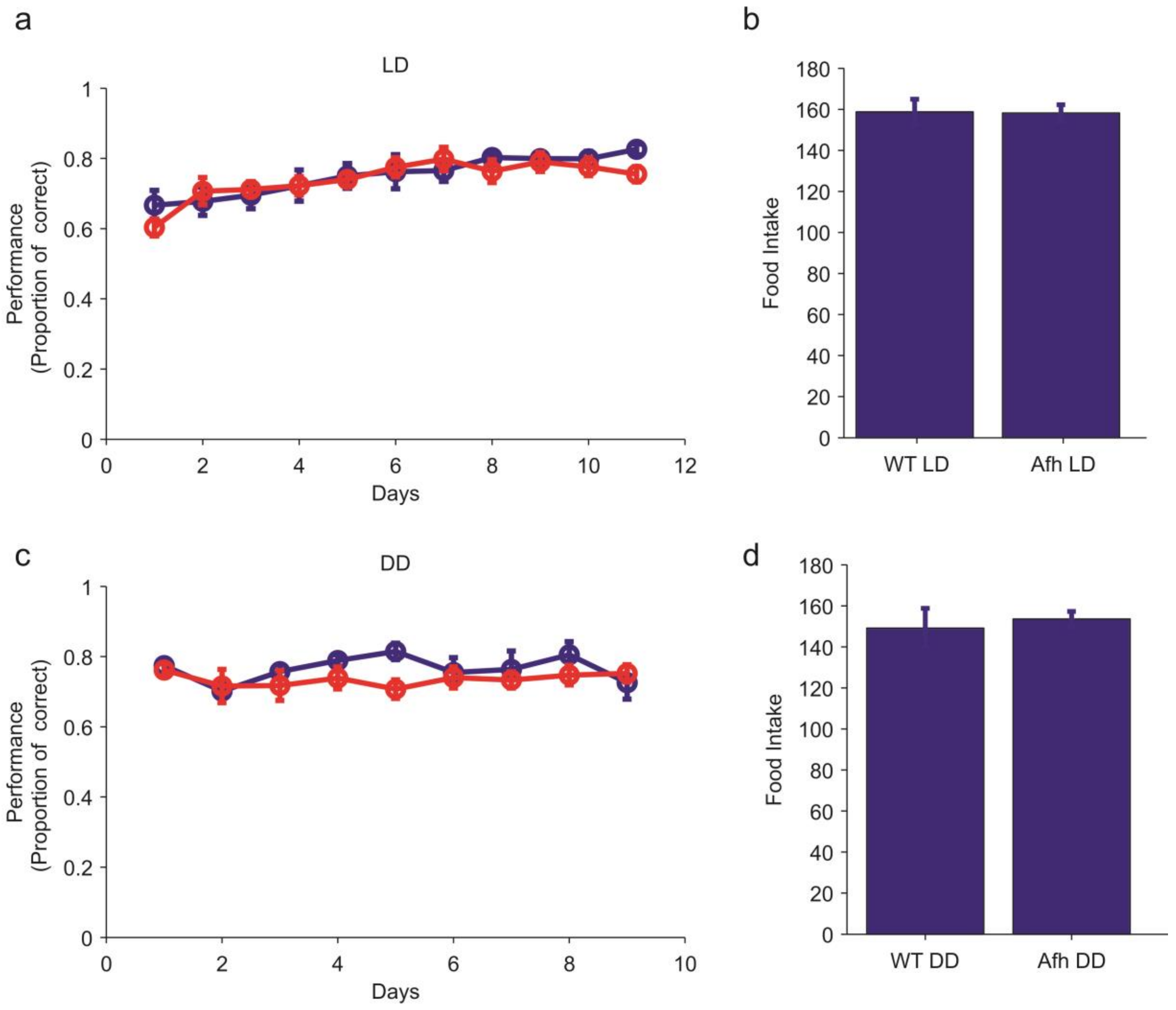

Figure S1. Behavioral performance during Switch Task. Learning curve over days of training for wild-type (blue) and *Afh* (red) mice as proportion of correct trials over all trials. Data are shown as mean ± SEM for LD (**a**) and DD (**c**) conditions. Average ± SEM of food intake as number of pellet eaten per day during the last 5 days of LD (**b**) and DD (**d**) conditions. These data refer to the task shown in Fig 5.

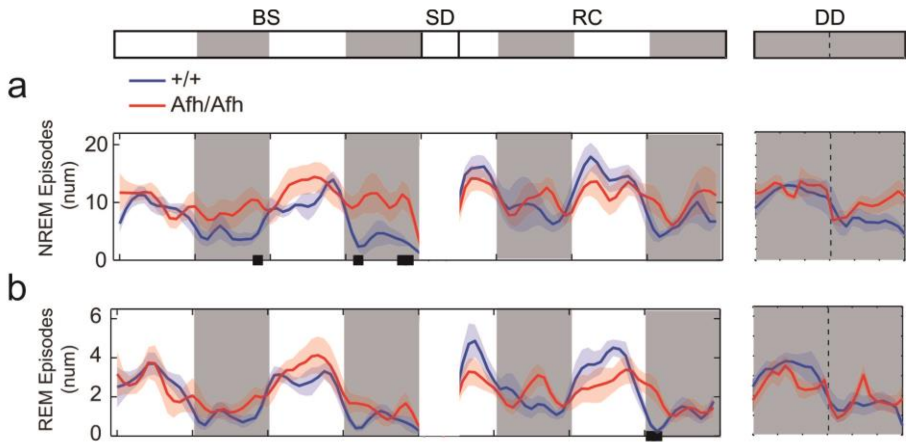

Figure S2. Sleep fragmentation is plotted as number of NREM (**a**) and REM (**b**) episodes during BS, RC after SD and DD condition. Grey shadow represents the dark phases and the black squares at the bottom of each graph indicate intervals where significant genotype differences were identified ($P < 0.05$, One-way ANOVA). Data are shown as mean ± SEM for wild-type (blue) and *Afh* (red) mutant mice.

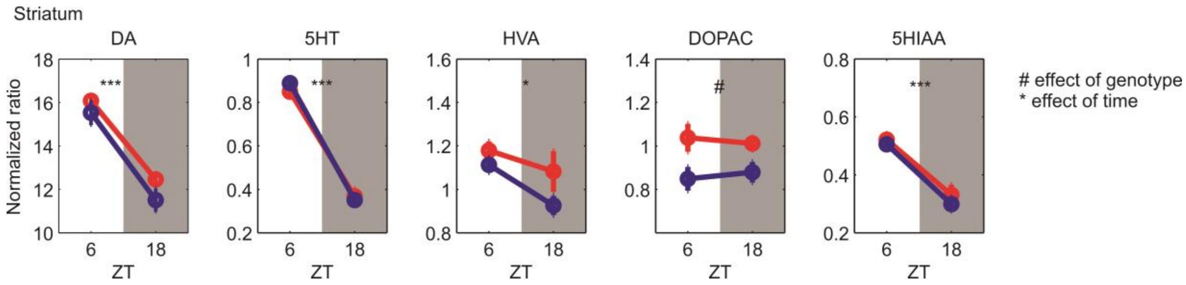

Figure S3. HPLC analysis of total tissue content. In total striatum, level of DA and HVA are significantly elevated during light phase (L) in comparison to dark phase (D) in both genotypes (***). The same tendency have 5HT and 5HIAA. Clear effect of genotype can be seen only for DOPAC level (#).

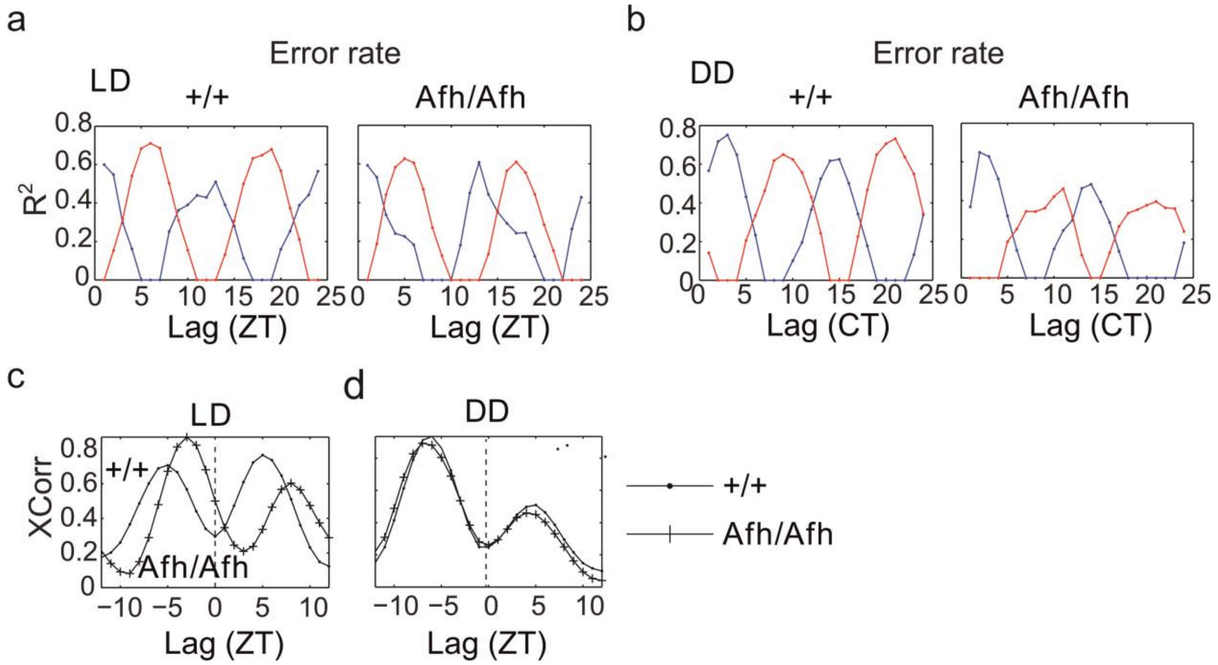

Figure S4 R^2 values for each lag obtained from the single regression of behavioral measure (Error rate) on Predictor 1 (Circadian activity, blue lines) and Predictor 2 (Process S, red lines) in LD (**a**) and DD (**b**). Comparison of the cross-correlogram between the red and blue lines in **a** and **b** for WT and *Afh* mutants for LD (**c**) and DD (**d**) conditions.

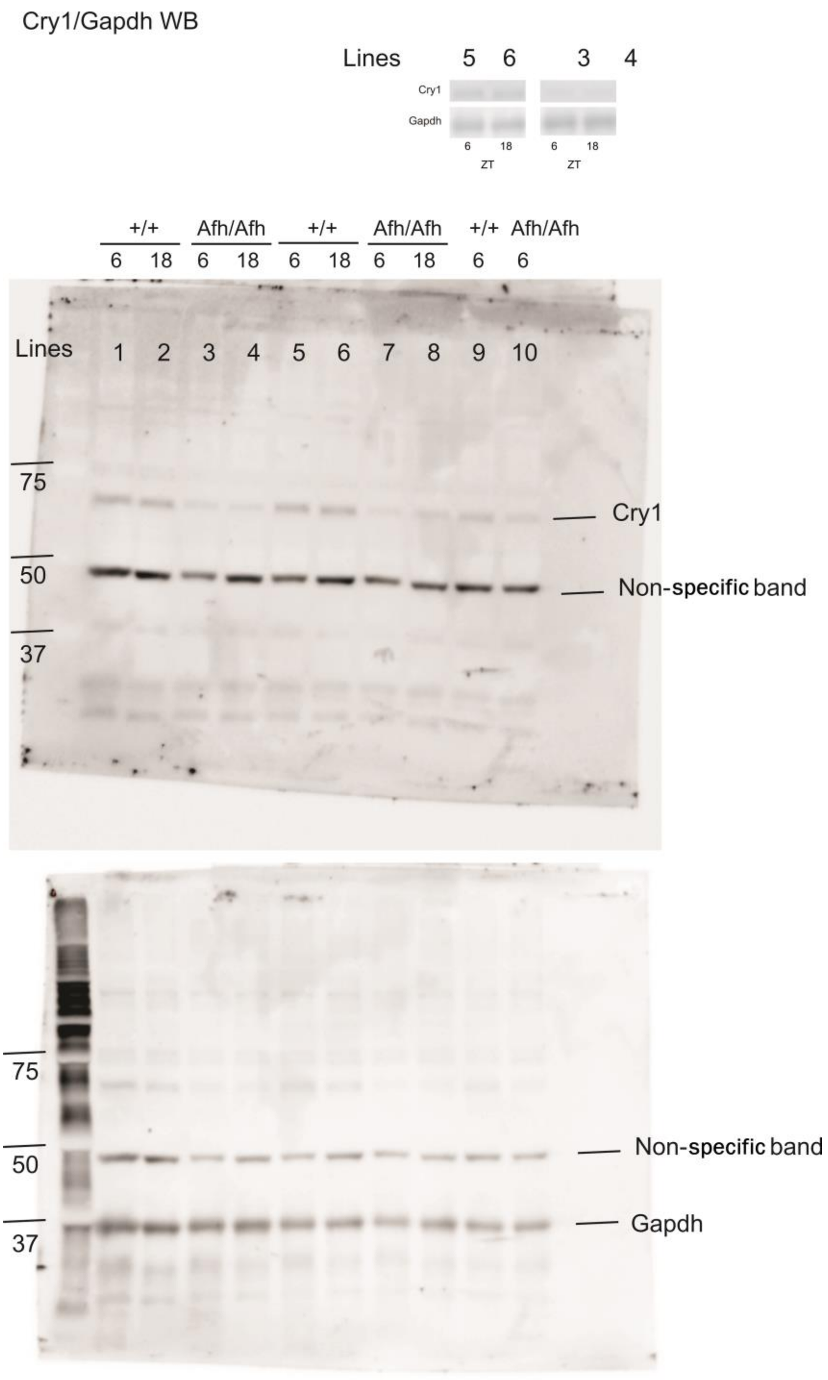

Figure S5 Full-length exposures of the same membrane showing Cry1 (upper panel) and Gapdh (lower panel) protein levels in striatum samples from +/+ and *Afh/Afh* animals at ZT6 and ZT18. 3 protein marker bands (37, 50 and 75kb) are depicted for reference. Both Cry1 (upper panel) and Gapdh (lower panel), together with non-specific bands, are indicated in the figure. Representative bands extracted from the

same membrane and same exposure are shown in figure 8e (specifically, bands 5-6 for +/+, and 3-4 for *Afh/Afh*).
